# Large positive in-plane magnetoresistance induced by localized states at nanodomain boundaries in graphene

**DOI:** 10.1038/ncomms14453

**Published:** 2017-02-15

**Authors:** Han-Chun Wu, Alexander N. Chaika, Ming-Chien Hsu, Tsung-Wei Huang, Mourad Abid, Mohamed Abid, Victor Yu Aristov, Olga V. Molodtsova, Sergey V. Babenkov, Yuran Niu, Barry E. Murphy, Sergey A. Krasnikov, Olaf Lübben, Huajun Liu, Byong Sun Chun, Yahya T. Janabi, Sergei N. Molotkov, Igor V. Shvets, Alexander I. Lichtenstein, Mikhail I. Katsnelson, Ching-Ray Chang

**Affiliations:** 1 School of Physics, Beijing Institute of Technology, Beijing 100081, China; 2 School of Physics, Centre for Research on Adaptive Nanostructures and Nanodevices, Trinity College Dublin, Dublin 2, Ireland; 3 Institute of Solid State Physics of Russian Academy of Sciences, Chernogolovka 142432, Russia; 4 Department of Physics, National Taiwan University, Taipei 10617, Taiwan; 5 Deutsches Elektronen-Synchrotron DESY, Hamburg 22607, Germany; 6 Institut für Experimentelle Physik, TU Bergakademie Freiberg, Freiberg 09596, Germany; 7 National Research University of Information Technologies, Mechanics and Optics, Saint Petersburg 197101, Russia; 8 MAX-lab, Lund University, Box 118, Lund 22100, Sweden; 9 Institute of Plasma Physics, Chinese Academy of Sciences, Hefei 230031, China; 10 Division of Industrial Metrology, Korea Research Institute of Standards and Science, Daejeon 305-340, South Korea; 11 Saudi Aramco Materials Performance Unit TSD, Research and Development Center, Dharhan 31311, Saudi Arabia; 12 Institut für Theoretische Physik, Universität Hamburg, Hamburg 20355, Germany; 13 Department of Theoretical Physics and Applied Mathematics, Ural Federal University, Mira Street 19, Ekaterinburg 620002, Russia; 14 Institute for Molecules and Materials, Radboud University, Heyendaalseweg 135, Nijmegen NL-6525AJ, The Netherlands

## Abstract

Graphene supports long spin lifetimes and long diffusion lengths at room temperature, making it highly promising for spintronics. However, making graphene magnetic remains a principal challenge despite the many proposed solutions. Among these, graphene with zig-zag edges and ripples are the most promising candidates, as zig-zag edges are predicted to host spin-polarized electronic states, and spin–orbit coupling can be induced by ripples. Here we investigate the magnetoresistance of graphene grown on technologically relevant SiC/Si(001) wafers, where inherent nanodomain boundaries sandwich zig-zag structures between adjacent ripples of large curvature. Localized states at the nanodomain boundaries result in an unprecedented positive in-plane magnetoresistance with a strong temperature dependence. Our work may offer a tantalizing way to add the spin degree of freedom to graphene.

## Abstract (web summary)

Owing to long spin lifetime and diffusion length, graphene holds promise for spintronics, yet it does not possess intrinsic magnetic properties. Here, the authors observe experimentally a large, positive magnetoresistance at graphene nanodomain boundaries.

Spin-transport electronics, or spintronics, is a subject of active international interest and investigation. Utilizing electrons' spin degree of freedom would give rise to new and more efficient forms of logic and storage devices^1^, as spin-polarized currents are capable of carrying more information than charge alone. Graphene is known for its extraordinary electronic properties, such as high electron mobility and tunable charge carrier concentration^2^,^3^. In addition, it also has the capacity for room-temperature spin transport, with propagation diffusion lengths of several micrometres, making it a highly promising material for spintronics^4^,^5^,^6^,^7^,^8^,^9^,^10^. Indeed, several graphene-based spin-logic devices have been proposed^11^,^12^. However, pristine graphene is diamagnetic and carbon does not possess *d* or *f* electrons. This means that inducing magnetic moments in graphene is non-trivial, and there have been many studies suggesting how this could be achieved, such as the introduction of vacancy defects^13^,^14^,^15^,^16^, doping with molecules or elements with high spin–orbital coupling^17^,^18^,^19^,^20^, and coupling with substrates or films^21^,^22^. Among the proposed methods, graphene with zig-zag edges or ripples are the most promising and intriguing ways to add the spin degree of freedom to graphene, as predictions suggest the zig-zag edges of graphene can play host to spin-polarized electronic states^23^,^24^,^25^,^26^,^27^,^28^, and spin–orbit coupling (SOC) can be induced and tuned by the curvature of the ripples due to hybridization of the *p*_*z*_ orbitals with *p*_*x*_ and *p*_*y*_ orbitals from the *σ*-band^29^. However, there has been no experimental support for these claims so far. On the other hand, it is well known that large-area graphene, fabricated by chemical vapour deposition^30^,^31^ or by vacuum synthesis on silicon carbide surfaces^32^,^33^, is frequently polycrystalline. Recent scanning tunnelling microscopy (STM)^31^,^32^,^33^ and transmission electron microscopy^34^,^35^ studies have shown that this graphene contains nanodomain boundaries (NBs), which can drastically modify the graphene's electronic transport properties^36^,^37^,^38^. It is believed that graphene with NBs will exhibit unique edge states with an unusual structure^23^,^24^,^25^,^26^,^27^,^28^,^39^. Specifically, the STM showed that graphene synthesized on SiC/Si(001) wafers contains NBs with a zig-zag structure on one side and an armchair structure on the other, which can induce a charge transport gap of at least 1.3 eV (refs 36, 37, 38). Thus, graphene synthesized on cubic-SiC is an ideal platform to investigate effects related to spin.

In this paper, we investigate the magnetoresistance (MR) of graphene synthesized on cubic-SiC(001). STM characterization indicates that a zig-zag structure forms on one side of our NBs and, moreover, ripples with large curvature are formed on both sides adjacent to the NBs. An unprecedented positive in-plane MR with a strong temperature dependence was observed. This positive MR decreases as the temperature is reduced from 300 to 100 K, reaching zero at 100 K, and increases again in the temperature range from 100 to 10 K. From non-equilibrium Green's function calculations, we show that the MR arises from the contributions of the NBs, which can explain the one-dimensional (1D) transport behaviour we observe at low temperatures. Above 100 K, the Zeeman effect leads to the inequivalent confinement of electrons with different spins from the two-dimensional (2D) sheet to the 1D NBs, which produces the positive MR observed at higher temperatures. This behaviour shows that it is possible to engineer new tunable electronic and magnetic nanostructures purely from graphene.

## Results

### Graphene growth and characterization

**Figure 1:**
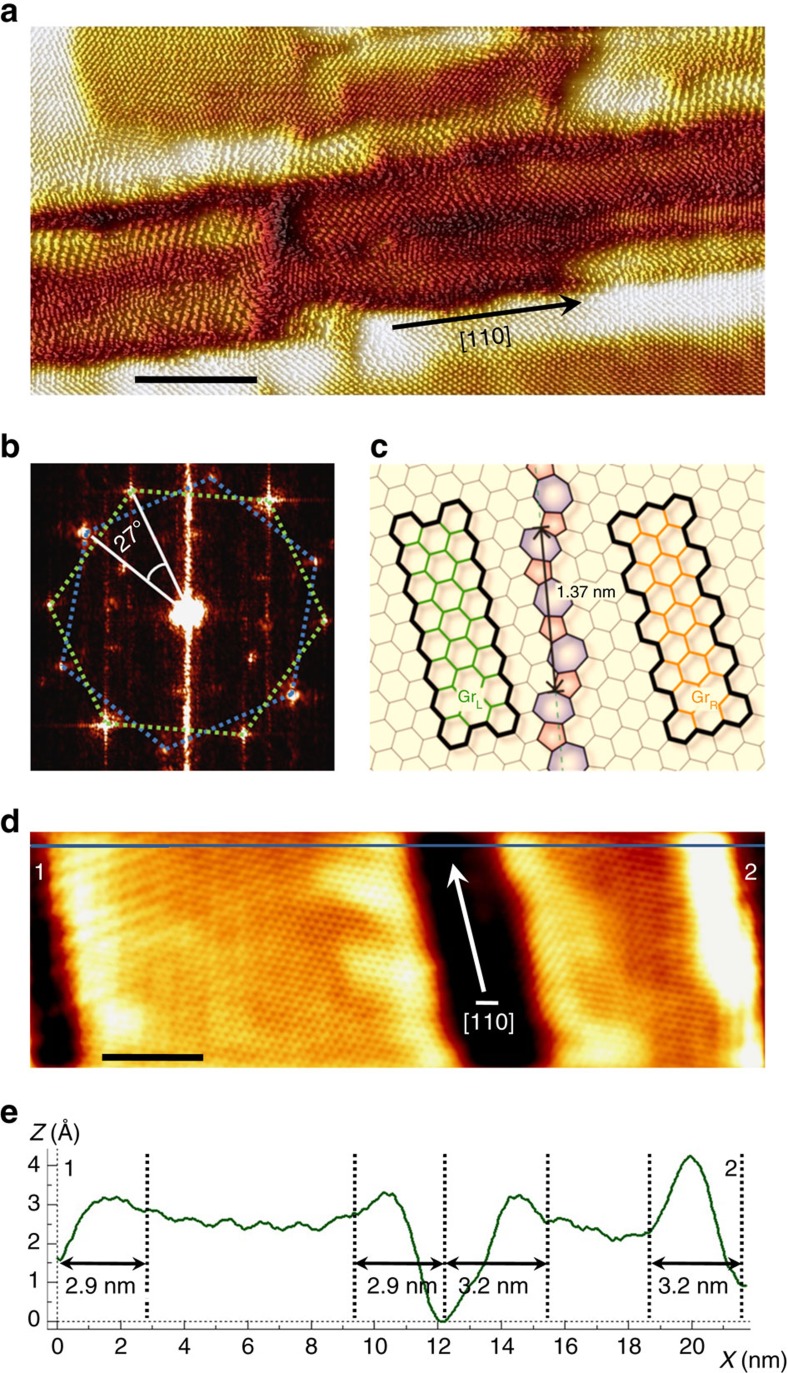
STM characterization of the trilayer graphene grown on SiC(001). (**a**) Atomically resolved STM image (30 × 15 nm^2^) of graphene on a cubic-SiC(001) surface showing the nanodomain structure with domains elongated in the [110] direction. Scale bar, 5 nm. (**b**) 2D-Fast Fourier Transform of the image with two 27°-rotated hexagons (dotted green and blue lines) overlaid. (**c**) Schematic drawing of the structure of NB to show zig-zag structure on one side and armchair structure on the other side. (**d**) Atomic-resolution STM image (21.8 × 6.9 nm^2^) of two nanodomains with the same lattice orientation elongated along the [1–10] direction. Scale bar, 3 nm. (**e**) Cross-section 1–2 from the image shown in **d**. The sizes of the ripples formed between the domains are indicated for clarity. The STM images were taken at *U*=22 mV and *I*=70 pA (**a**) and *U*=−20 mV, *I*=100 pA (**d**).

Figure 1a shows a typical atomically resolved STM image of the trilayer graphene synthesized on SiC(001). The image shows that the graphene overlayer consists of nanometre-scale domains, which have bright boundaries oriented approximately along the one of the two orthogonal <110> directions. The nanodomains have lateral dimensions ranging from a few to 40–50 nm. A Fast Fourier Transform of the STM image (Fig. 1b) reveals two preferential orientations of the graphene lattices in the nanodomains, which are rotated by 27° relative to one another, that is, ±13.5° from the [110] direction. High-resolution STM images of the NBs between the 27°-rotated lattices (for example, Supplementary Fig. 1c) show that, in most cases, the NBs are rotated by 3.5° relative to the one of the two orthogonal <110> directions leading to an asymmetrical rotation of the graphene lattices in the neighbouring domains. They are rotated by 17° clockwise and 10° counterclockwise relative to the NBs, and there is a zig-zag structure on one side and an armchair structure on the other side of the NBs (Fig. 1c)^36^. An atomic-resolution image of two nanodomains with non-rotated graphene lattices is shown in Fig. 1d. This image demonstrates the extreme distortion of the overlayer near the dark regions appearing as NBs. The graphene sheet in these areas is buckled first upwards and then downwards, forming semi-tubes, aligned along the [1–10] direction, with typical apparent diameters of several nanometres, as the cross-section in Fig. 1e illustrates. For the image shown in Fig. 1d the widths of the [1–10]-directed semi-tubes are in the range of 2.6–3.2 nm, although in other surface regions their widths vary between 2 and 5 nm (for example, Fig. 1a and Supplementary Fig. 1c). Thus, the NBs are a combination of both ripples and graphene grain boundaries. The grain boundaries in our study consist of distorted heptagons and pentagons, and zig-zag and armchair edges develop from them. Further details can be found in Supplementary Note 1 and elsewhere^36^. A high-resolution STM image taken in the middle of a nanodomain (inset of Supplementary Fig. 1c) reveals a honeycomb pattern deformed by atomic-scale rippling typical of quasi-freestanding graphene^40^.

### Positive in-plane MR in hall-bar geometry

**Figure 2:**
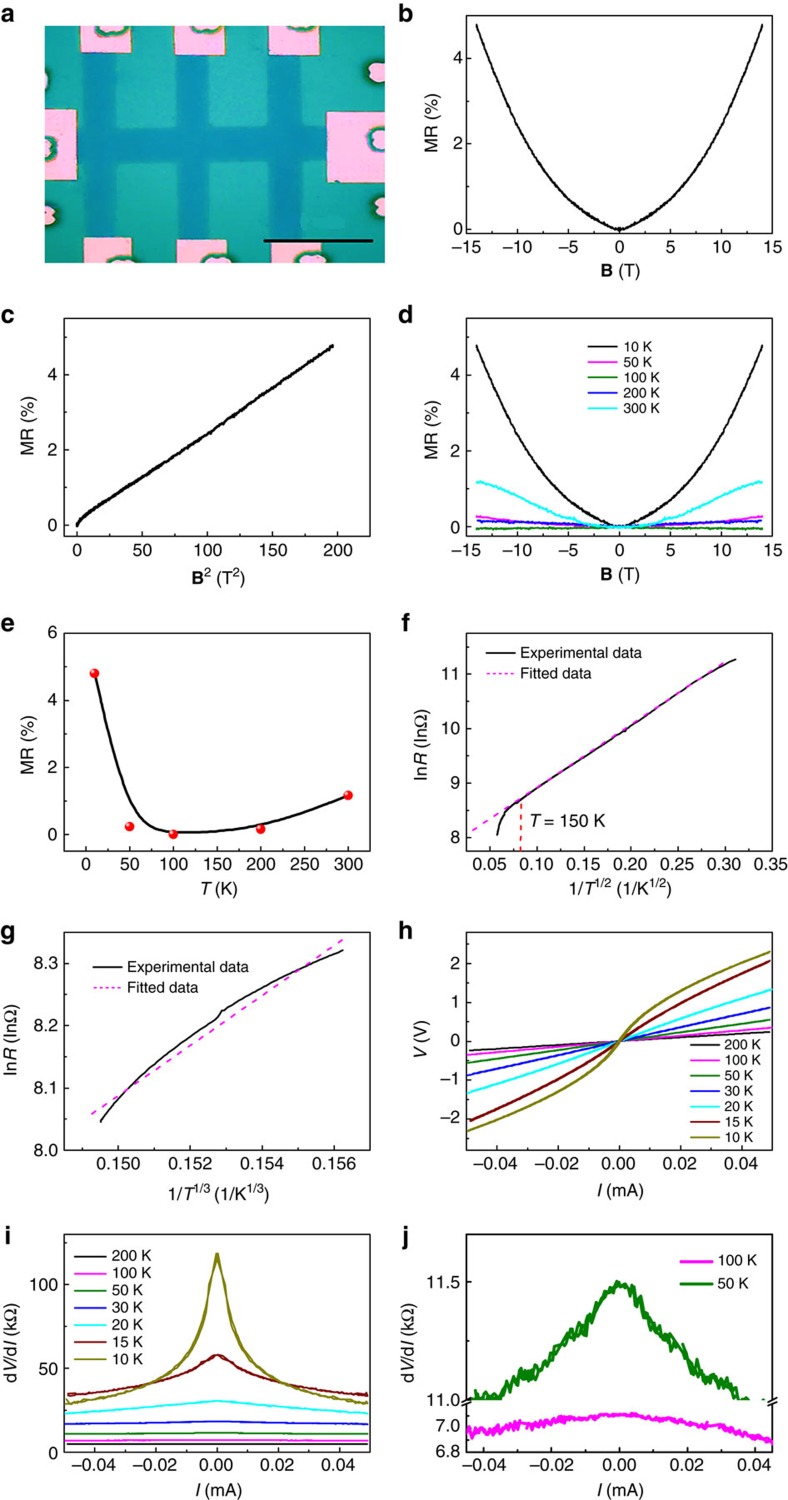
MR and *I-V* characterization of graphene on SiC. (**a**) Typical optical image of the graphene Hall bar device. Scale bar, 40 μm. (**b**) MR curve measured at 10 K with an in-plane magnetic field. (**c**) MR for an in-plane magnetic field as a function of **B**^2^. (**d**) Temperature dependence of MR curves measured with an in-plane magnetic field along the current direction. (**e**) MR ratio for an in-plane magnetic field as a function of temperature. (**f**) Resistance *R* as a function of temperature *T* in the low temperature range 10–150 K. The low-temperature behaviour of ln(*R*) can be fitted as a straight line with respect to the variable 1/*T*^1/2^, indicating a 1D channel behaviour in the VRH model. (**g**) At high temperatures, ln(*R*) shows a clearly different behaviour and can be fitted better with the function 1/*T*^1/3^ up to 300 K, suggesting 2D VRH transport. *I–V* curves (**h**) and the corresponding d*V*/d*I* curves (**i**,**j**) measured at a variety of temperatures.

Figure 2b shows the MR curve measured at 10 K with an in-plane magnetic field, for a graphene hall-bar device as shown in Fig. 2a. From Hall measurements it was determined that the electron mobility in our samples is ∼250 cm^2^ V^−1 ^s^−1^ at 10 K and 60 cm^2^ V^−1 ^s^−1^ at 300 K, and the corresponding current densities are 2 × 10^12^ cm^−2^ at 10 K and 2 × 10^13^ cm^−2^ at room temperature. This value for electron mobility is notably less than the ∼1,000 cm^2^ V^−1 ^s^−1^ observed for trilayer graphene prepared by mechanical exfoliation^41^. It can be concluded that the presence of NBs decreases the electron mobility of graphene prepared on SiC and suggests that a significant number of electrons are confined to the NBs at low temperature. Remarkably, a positive MR of 5% is observed. As an archetypical two-dimensional (2D) material, all the carriers are expected to move freely inside the pristine graphene and the MR should be zero due to the weak interaction between the electrical current and the in-plane magnetic field^42^. We plot the MR as a function of field squared (**B**^2^) in Fig. 2c. It was found that the MR curve for an in-plane field shows a linear **B** dependence at low field and a quadratic **B**^2^ dependence at high field. To rule out the possibility that this effect might be due to misalignment between the magnetic field and graphene plane, Supplementary Fig. 2 shows the angular-dependent resistance of a device measured at 10 K under a magnetic field of 14 T. The maximum and minimum resistances appear at 0° and 90°, respectively. Supplementary Fig. 3 further shows the MR measured at 10 K, for a selection of field orientations, from the magnetic field in an out-of-plane (perpendicular to graphene plane, *θ*=0°) to an in-plane (aligned with the current, *θ*=90°) configuration, where *θ* is the angle between the direction of the magnetic field and the direction normal to the graphene plane. The MR exhibits a strong dependence on *θ* and the MR ratio increases with decreasing *θ*. For *θ*<60°, the MR ratio is always negative because of a reduction in scattering at the NBs. Therefore, misalignment between magnetic field and graphene plane can be ruled out as the source of the effect. A similar phenomenon was observed at other temperatures (Supplementary Fig. 4). Figure 2d shows the temperature dependence of MR curves measured with an in-plane field. Remarkably, the MR decreases with falling temperature and reaches zero at 100 K (Fig. 2e). Further decreasing the temperature causes the MR ratio to increase once again. The positive MR observed could be due to the presence of the ripples formed at the NBs, which bend the graphene layer almost perpendicular to the substrate and result in a conventional ordinary MR from the out-of-plane cyclotron motion^42^. However, even with a strong magnetic field on the order of several Tesla, the cyclotron radius is as large as tens of nanometres, significantly larger than the diameters of the ripples (2–5 nm). Therefore, the positive MR being due to ordinary MR from out-of-plane cyclotron motion can be ruled out. It can also be ruled out that any of these features arise due to electrical contributions by the silicon carbide substrate or the formation of alloys on the silicon carbide substrate^43^. As the resistance of the bare SiC is at least three orders of magnitude greater than the graphene, the influence of the substrate can be ignored (Supplementary Fig. 5 and Supplementary Note 2). Moreover, our systematic studies of the graphene/SiC(001) systems by various surface science techniques have verified that no alloys were formed on the SiC substrate and the formed structures consisting of quasi-freestanding few-layer graphene overlayers weakly interacting with the substrate^44^,^45^, were free of contaminants (Supplementary Note 3). Another possibility is that spin scattering at magnetic/nonmagnetic impurities could results in a positive MR. Supplementary Fig. 6a shows the MR curves of graphene measured at various temperatures with the magnetic field applied normal to the graphene plane. A negative MR is observed, indicating a possible weak localization effect. We have extracted the dephasing rates at various temperatures and summarized them in Supplementary Fig. 6b^46^. It shows a nonlinear response with temperature. Thus, spin scattering at magnetic impurities can be ruled out (Supplementary Note 4)^46^. Moreover, since the spin dephasing time due to impurities is on the order of 10 ps and if we consider a moderate diffusion constant *D*∼100 cm^2 ^s^−1^ (ref. 47), the mean free path is in the range of about a few tens of nm to hundreds of nm (consistent with those estimated in Supplementary Fig. 6b), which is larger than the average distance between NBs in our sample (∼30 nm). Therefore, even if magnetic impurities were to contribute to the resistance change (if they exist at all), we expect the NBs will still have a much larger influence. We would like also to stress that our atomic-resolution STM studies show that the graphene nanodomain structure is uniform throughout millimetre-sized graphene/SiC/Si(001) samples. Almost no single-atomic impurities were observed on the surface. In addition to this, using our non-equilibrium Green's function (NEGF) calculations that consider the NB arrangement, we will show that in-plane and out-of-plane fields produce positive MR and negative MRs, respectively, and this is consistent with the experimental data. Thus, a reasonable explanation for the positive in-plane MR comes from the intrinsic properties of the NBs themselves.

To investigate the effect of the NBs on the magneto-transport properties, Fig. 2f,g plots the resistance as a function of temperature. The resistance of our sample increases monotonically with decreasing temperature, from 300 to 10 K, and shows non-metallic behaviour. At *T*<150 K, the conductive mechanism can be described well using a 1D variable-range hopping (VRH) model^48^ 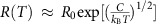, where *R*_0_ and *C* are the fitting constants, indicating that the transport below 150 K is restricted to the NBs. In contrast to the low-temperature data, the *R–T* curve above 150 K has a different slope and is fitted better by the cube root expression 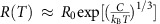, suggesting 2D transport. This means that carriers can cross the NBs in the high-temperature regime. The deviation of the fitting from the 2D transport model at 300 K is due to thermally assisted hopping, which is allowed along the ripples in the *z* direction at high enough temperatures. To verify the nature of the transport mechanisms below and above 150 K, Fig. 2h–j shows the *I–V* curves and the corresponding d*V*/d*I* measured at different temperatures. Above 150 K, the current increases linearly with applied voltage, clearly highlighting the 2D transport mechanism in this temperature range. The linear *I–V* curves also indicate good Ohmic contact between the electrodes and graphene. Interestingly, below 100 K, nonlinear *I–V* curves were observed and the d*V*/d*I* shows a maximum at *I*=0 mA, indicating the presence of a charge transport gap below 100 K. At low temperatures, the NBs have 1D localized edge states^49^ because they form a flat band, which can effectively confine the states^50^. The 1D localized edge states provide a platform for the thermally activated transport, and thus the carriers are transported along the NBs^36^, hence the fit with the 1D VRH model. As the temperature increases, the thermal energy allows hopping between the pristine graphene and NBs, which causes the dimension of VRH transport to spread to essentially 2D.

### Theory of MR of graphene with a single NB

**Figure 3:**
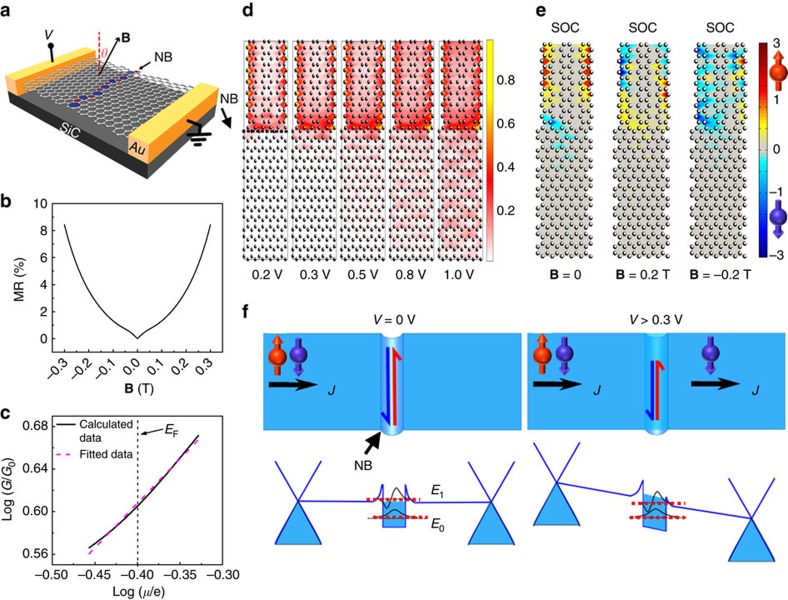
MR and spin-filtering effect of graphene containing a single NB. (**a**) Schematic drawing of the model used. (**b**) MR of graphene containing a single NB calculated with an in-plane magnetic field from using the NEGF method. (**c**) Calculated conductance 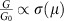
as a function of *μ* under a 4 T field with 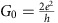. The best fit of *σ*(*μ*)∝*μ*^*α*^ gives *α*≈0.839<1. This sublinear dependence is consistent with the positive MR criterion from the Kubo–Greenwood formula. (**d**) Charge distribution at different bias voltages calculated from NEGF simulations indicating the presence of a transport gap below 0.3 eV and a high charge density along the NB. The bias voltage is applied from the top to the bottom and the current passes through the NB. The colour intensity indicates the relative magnitude of charge density. (**e**) Spin density distribution in the *z* direction (perpendicular to graphene plane) under a bias voltage of 0.4 V calculated from NEGF simulations to demonstrate the spin-filtering effect due to the localized state of a NB and SOC of 0.1 meV at ripples. The sign indicates the orientation of the spins and the unit of intensity is 7 × 10^−26^ *ħ*m^−2^. (**f**) Schematic illustration of electrical transport and spin-filtering effect due to localized state of NBs and SOC at ripples.

In general, according to the Kubo–Greenwood formula^51^, the conductivity *σ* under an in-plane field for massless Dirac fermions in pristine graphene should give a positive Δ*σ*(**B**) and thus a negative MR^52^. Contrary to this, the positive MR of graphene with NBs under an in-plane magnetic field shown here implies a negative Δ*σ*(**B**), which requires a sublinear behaviour *σ*(*μ*)∝*μ*^*α*^ with 0<*α*<1, where *μ* is the chemical potential. Details of the calculation can be found in the Supplementary Note 5. However, the MR of graphene with NBs is not only positive but also shows a linear dependence on **B** at low fields and a quadratic **B** dependence at high field. Moreover, a rather strong temperature dependence is also observed. The most reasonable explanation for the positive MR comes from the intrinsic properties of the NBs themselves and that the carriers have different transport mechanisms below and above 150 K due to the presence of the NBs. Here we explain how the NBs can modify the Δ*σ*(*μ*) and the MR of graphene by calculating the transport properties of graphene containing a single NB based on a NEGF calculation and the Landauer–Keldysh formalism^53^. Details of the simulation can be found in the Methods section and a schematic of the simulated system is provided in Fig. 3a. Figure 3b shows the calculated MR of graphene containing a single NB with an in-plane magnetic field. A positive MR with **B** dependence at low field and **B**^2^ dependence at high field is predicted by the calculation in agreement with what was experimentally observed. We simulated different lengths of the pristine graphene while maintaining the dimensions of the NB and found that the MR dependence remains exactly the same (Supplementary Figs 7 and 8). Figure 3c plots the calculated *σ*(*μ*) as a function of *μ* for **B**=4 T, confirming that for graphene with NBs, *σ*(*μ*) has a sublinear behaviour *σ*(*μ*)∝*μ*^*α*^ with *α*≈0.839. Thus, we can conclude that the positive MR under an in-plane magnetic field at low temperatures arises predominately from the NB itself. Figure 3d shows the calculated charge density distribution of the device under various bias voltages. There is an obvious charge density accumulation at the NB, and when the bias is increased to a value of 0.5 V the charge density begins to spread across to the NB. The phenomenon is consistent with our experimental observations^36^. Moreover, the charge density along the NB is greater than that in the pristine graphene, which clearly demonstrates the 1D transport properties of the NBs at low bias voltages. This is also in agreement with STM studies of a NB in graphene on Ni, where the defect was observed to act as a quasi-1D metallic wire^54^. Furthermore, our graphene additionally has large curvature at the ripples (Supplementary Fig. 9 and Supplementary Note 6), which is quite similar to a carbon nanotube with small radius. It is reported that the SOC of a carbon nanotube critically depends on its radius, and can be as high as 3.5 meV (refs 55, 56). However, there are some boundary regions where the radius of curvature is greater than ∼2.5 nm, which would mean that the SOC could be an order of magnitude weaker. To investigate the spin-dependent transport across the NBs and in particular considering the case with a weaker SOC associated with a larger radius of curvature, Fig. 3e shows the calculated spin density distribution of the device under a bias voltage of 0.4 V. The SOC is set to be 0.1 meV to reflect the regions that have larger radii of curvature. One can see that under a bias voltage of 0.4 V, only electrons with a particular spin can across the NBs, indicating that NBs with ripples can work as spin filters and SOC at ripples gives rise to spin-dependent energy splitting. Moreover, when an in-plane magnetic field is applied perpendicular to the NBs, fewer electrons can cross the NBs, implying a positive MR, which is consistent with the MR calculation. It is known that disordering effects may exist within a regular sample and several possible effects can be involved. We investigated, for the same SOC strength, the following disordering effects: variation of length; disorder within a single NB; and the orientation of the magnetic field (Supplementary Figs 10–13 and Supplementary Note 7). It is shown that the relative strengths of the spin filter and confinement effects are only marginally influenced and that the fundamental phenomenon is still observed. Note that a recent theoretical study suggested that a graphene boundary itself with a specific configuration may induce a spin-filtering effect due to the asymmetry between up and down spins^57^. However, we would like stress that the grain boundaries in our study are quite different from those in ref. 57. The grain boundaries in our study consist of distorted heptagons and pentagons, and the ripples are formed to relieve in-plane compressive strain. Moreover, more recent theoretical studies investigated the influence of localized magnetic moments at the edge on the transport properties and suggested that localized magnetic moments could be screened by the polarized current under bias^58^,^59^. Therefore, the spin-filtering effect in our study is expected to be mainly due to SOC as reported for other curved graphene systems.

To estimate the transition of the energy scaling from 1D to 2D, once the temperature is sufficient, we can treat the graphene with a NB system as a quantum well that can be overcome by either a bias voltage or thermal excitation. The NB quantum well has a depth of 0.4–1 V, as suggested by *I–V* characterization^36^ and a width of ∼6 nm (Fig. 1e). In our sample, there are several aligned NBs separated by an average distance of 12–15 nm. The energy difference between the ground state (*E*_0_) and the first excited state (*E*_1_) for each quantum well will thus be on the order of 0.1 eV as estimated from the finite-depth quantum well confinement. Once energized by an applied bias or thermal agitation, electrons can jump to the first excited state, and the wave functions of the localized edge states associated with the NBs begin to smear out more readily (Fig. 3f). When the overlap between the wave functions of neighbouring quantum wells is large enough, the electrons can overcome their NB confinement, and can thus traverse the entire 2D graphene plane. Moreover, different spins trapped at NBs will have different potential depths due to the SOC at the ripples. Therefore, the energy differences between the ground state and the first excited state are also spin-dependent. Once the temperature is raised to 100 K, electrons regardless of their spin state can jump to the excited states as the difference in energy between the spin states is small compared with *k*_B_*T*. The confinement at the NB is weak at 100 K and thus the positive MR from the confinement to the NB itself goes to zero. At higher temperatures, most electrons jump to the excited states and the overlap between the wave functions of the NBs creates paths for 2D transport. However, once an in-plane magnetic field is applied, Zeeman splitting raises the energy levels for one of the spins, making it more difficult for electrons with this particular spin to reach the excited state. Therefore, electrons with one spin direction become more localized in the 1D NB under an in-plane magnetic field. This separation of the spins by the Zeeman effect reduces transport and hence produces a positive MR at temperatures higher than 100 K. We therefore conclude that below 100 K the MR results from the NBs alone, while above 100 K the MR comes from spin confinement, that is, the reduction of the 2D transport for electrons with a particular spin direction that are confined to 1D.

### Transport properties of graphene with nano-gap contacts

**Figure 4:**
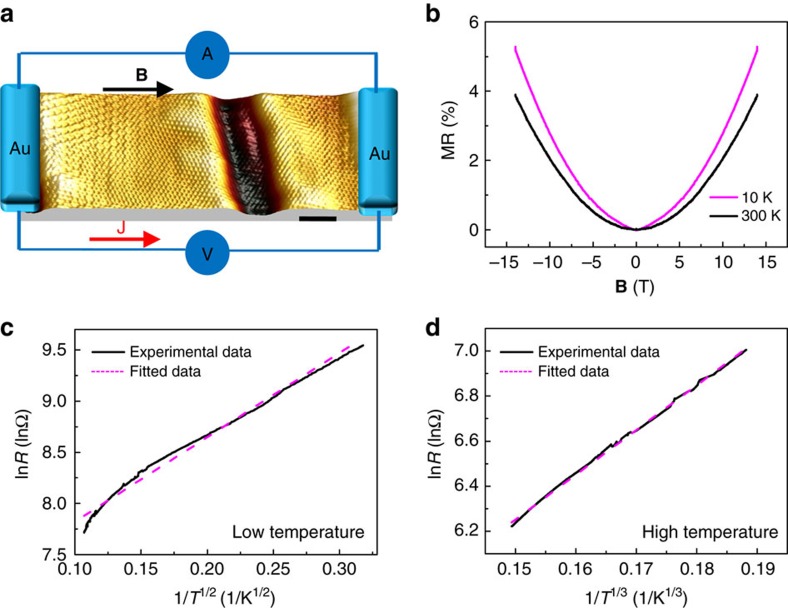
MR and transport properties probing one to two NBs. (**a**) Schematic drawing of the graphene devices with nano-gap contacts. The spacing of the nano-gap was ∼28 nm. Scale bar, 3 nm. (**b**) MR curves of the nanocontact device measured at 10 and 300 K, with the magnetic field in the graphene plane aligned along the direction of current flow (*θ*=90°). (**c**) The log resistance ln(*R*) can be fitted as a straight line with respect to 1/*T*^1/2^ in the low temperature range 10–80 K, indicating 1D transport from the VRH model. (**d**) At higher temperatures up to 300 K, the ln(*R*) behaviour is different and is best fitted as a function of 1/*T*^1/3^, suggesting 2D VRH transport.

To further investigate the effect of NBs on the transport properties and confirm the theoretical explanation, we fabricated several devices with nano-gap contacts. Figure 4a shows a schematic drawing of a typical nano-gap device. The gap between the electrodes was ∼28 nm, thus one to two NBs were measured within the gap and the voltage was applied perpendicular to the NBs. Figure 4b shows MR curves measured at 10 and 300 K with an in-plane magnetic field. A positive MR was observed at both temperatures, which is a confirmation that the positive MR is due to the NBs. Figure 4c,d plots the resistance as a function of temperature under zero magnetic field and non-metallic behaviour was observed. The plots were also fitted with a *d*-dimensional VRH model, 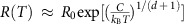. Similar to cases for the micrometre-scale devices shown in Fig. 2, the *R*–*T* curves can be fitted well using the 1D VRH model at low temperature (Fig. 4c) and using the 2D VRH model at higher temperatures (Fig. 4d). The two mechanisms at high and low temperatures are similar to those discussed above, but the transition temperature is ∼80 K. Compared with Fig. 2, the resistance for the nano-gap device at high temperature displays more 2D-like behaviour because carriers can pass through the whole small nanometre-scale area between the electrodes more easily. For the micrometre-size device in Fig. 2, more energy or a higher temperature is required to overcome the NBs and produce efficient 2D transport. The thermally supplied energy must be sufficient to allow transport through at least two NBs in order for this to occur, corresponding to an approximate doubling of the energy, which is associated with the increased transition temperature for the micrometre-scale system with respect to that in the nano-gap system. In contrast, at low temperature, the *R*–*T* curve for the nanometre-size device can be fitted reasonably well by 1/*T*^1/2^, giving evidence of 1D transport along a NB (Fig. 4c); however, this fit not as good as for the micrometre-scale case (Fig. 2f). The reason is that when only one or two NBs are measured, since the voltage is applied perpendicular to the NB, the electrons must tunnel through the NBs. If we apply voltage along NBs, a clear 1D channel behaviour is observed (Supplementary Fig. 14).

## Discussion

In conclusion, we have presented a magnetic transport study of trilayer graphene synthesized on cubic-SiC(001). Under an in-plane field, we observed a positive MR with a strong temperature dependence. Using NEGF calculations combined with experimental data, we have shown that the NBs with ripples have the potential to work as a spin filter and can result in a positive MR at low temperature. Moreover, our work suggests that graphene with NBs has localized states and large spin–orbit interaction at the ripples. The confinement of electrons of a particular spin direction from 2D to 1D NBs by the Zeeman effect is responsible for the positive MR observed at high temperatures.

## Methods

### Sample preparation and characterization

The uniform trilayer graphene was fabricated on a cubic-SiC thin film with a thickness of ∼1 μm grown on on-plane Si(001) wafers using Si-atom sublimation followed by high-temperature surface graphitization in the ultra-high vacuum (UHV) chamber of a room-temperature GPI-300 scanning tunnelling microscope^35^,^36^. The base pressure in the STM chamber was kept below 6 × 10^−11^ Torr. The pressure during the graphene synthesis at sample temperatures of 1,300–1,350 °C did not exceed 4 × 10^−10^ Torr. STM characterization of the graphene trilayer synthesized on SiC(001) was carried out *in situ* at room temperature using single crystalline W[111] and W[001] tips prepared using chemical etching and UHV sharpening. Low energy electron microscopy (LEEM) measurements were done using a SPELEEM microscope (Elmitec GmbH) installed on I311 at the MAX laboratory in Sweden. Before the LEEM characterization, the trilayer graphene/SiC(001) samples were annealed in the microscope's UHV chamber at temperatures ranging from 600 to 1,000 °C to remove possible contamination.

### Device fabrication

The field effect transistor Hall bar structure was fabricated by electron beam lithography using a single-layer negative tone resist ma-N 2403 supplied by MicroChem Corp, followed by O_2_ plasma etching for 60 s (8 W at *P*=20 mTorr). The nanocontact fabrication was carried out by electron beam lithography using single-layer-positive tone resist poly(methyl methacrylate) (PMMA) supplied by MicroChem Corp. After development, thick metal contacts consisting of Ti (5 nm)/Au (45 nm) were deposited through e-beam evaporation.

### Electron transport calculations

A tight-binding model was used to describe the system with a nearest neighbour hopping energy *t* equal to 2.7 eV, and the system Hamiltonian is^60^,


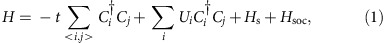


where *C*_*i*_^†^ *(C*_*i*_) creates (annihilates) an electron at site *i* and *U*_*i*_ is the onsite energy at site *i*. The onsite energy in the graphene system is the energy of the *P*_z_ orbital due to position variance^61^. Since a Stone–Wales defect causes only a small deviation in the bond length^62^,^63^,^64^, we can therefore assume that all of the hopping energies are the same and the onsite energy can be set to zero because all the carbon atoms are assumed to be equidistant. More accurate *t* and *U*_*i*_ values can be calculated by taking into account the position variance caused by NBs or their substrate and so on^65^. The third term in the Hamiltonian is the Zeeman term, 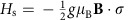, where *g* is the Landé g-factor, 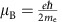, the Bohr magneton, **B** is the external magnetic field and *σ* is the Pauli matrix. The last term in the Hamiltonian is Rashba spin–orbital coupling, induced by the ripple near the NB, 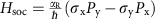, where *α*_R_ is the SOC strength. In the simulation we consider that the NB and its nearby graphene two-layer neighbours both have Rashba SOC. Note that no phonons were involved in our calculations. We use the NEGF formalism to calculate magnetoresistence^53^,^66^. The transmission function between the leads is evaluated by


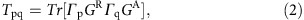


where *G*^R^ (*G*^A^) is the retarded (advanced) Green's function of the system^53^. The lead broadening function *Γ*_p(q)_ is defined as 
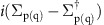
, where *Σ*_p(q)_ is the self-energy of the lead. From the transmision *T*(*E*), we can get the conductance *G*(*E*)=*G*_0_*T*(*E*), where 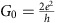 and also 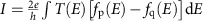 where *f*_p(q)_ is Fermi distrbution of each lead. In our MR calculations, the bias *V* is fixed at 0.4 V and the resistance is obtained by *R*(**B**)=*V*/*I*(**B**). The conductivity *σ*(*μ*) is proportional to the transmission and can be calculated from *σ*(*μ*)∼*T*(*E*=*μ*). The charge density is calculated by the local occupation number at each site. The occupation number operator at site *i* is 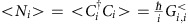, where *G*^*<*^ is the lesser Green's function whose formula is *G*^*<*^*(E)=G*^R^*(E)Σ*^*<*^*(E)G*^A^*(E)*, where 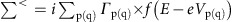. The local spin occupation number at site *i* is 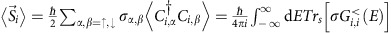, where *σ*=(*σ*_x_, *σ*_y_, *σ*_z_) are the Pauli matrices, and *Tr*_s_[…] denotes the trace in the 2 × 2 spin −½ space.

### Data availability

The data that support the findings of this study are available from the corresponding author upon request.

## Additional information

**How to cite this article:** Wu, H.-C. *et al*. Large positive in-plane magnetoresistance induced by localized states at nanodomain boundaries in graphene. *Nat. Commun.* **8,** 14453 doi: 10.1038/ncomms14453 (2017).

**Publisher's note:** Springer Nature remains neutral with regard to jurisdictional claims in published maps and institutional affiliations.

## Supplementary Material

Supplementary Information Supplementary Figures, Supplementary Notes and Supplementary References

Peer Review File
