# Supplementary material for: Large positive in-plane magnetoresistance induced by localized states at nanodomain boundaries in graphene
Source: Nat Commun. 2017 Feb 15;8:14453. doi: 10.1038/ncomms14453 (PMC5316875; doi:10.1038/ncomms14453)
Supplement: Supplementary Information — Supplementary Figures, Supplementary Notes and Supplementary References [file ncomms14453-s1.pdf]

Supplementary Figures

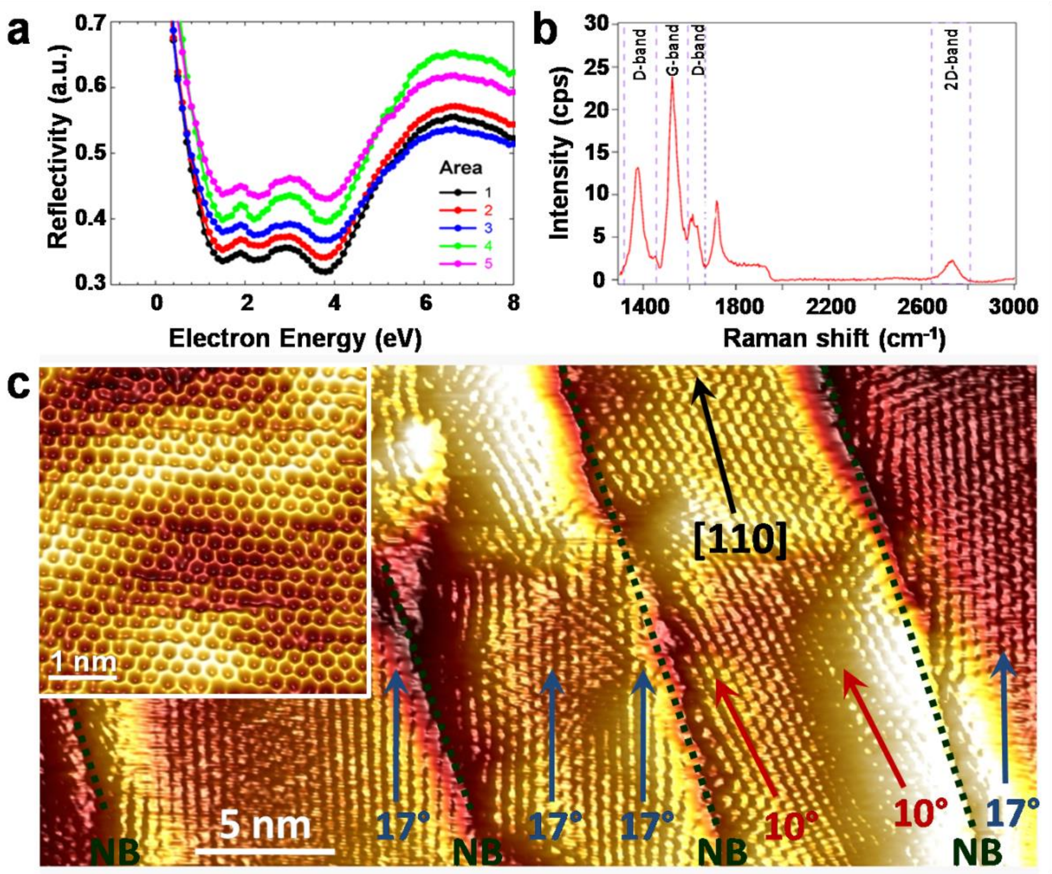

Supplementary Figure 1 | Characterization of graphene on SiC. (a) Electron reflectivity curves acquired in a 7 eV energy window to demonstrate the layer thickness of graphene. (b) Typical Raman spectrum of graphene on a SiC substrate with an excitation photon energy of 2.33 eV, indicating the graphene grown is trilayer graphene with domain boundaries. (c) Atomically resolved STM image of graphene on a cubic-SiC(001) surface showing the system of 27°-rotated nano-domains elongated in the [110] direction and the atomic structure of the domain boundaries. The domain lattices are rotated by 17° clockwise and 10° counter clockwise relative to the nanodomian boundaries (NBs). Inset: 5×5 nm^2 STM image showing a honeycomb lattice and atomic scale ripples characteristic of free-standing graphene. The images were measured at U=-20 mV, I=60 pA (c) and U=22 mV, I=65 pA (inset).

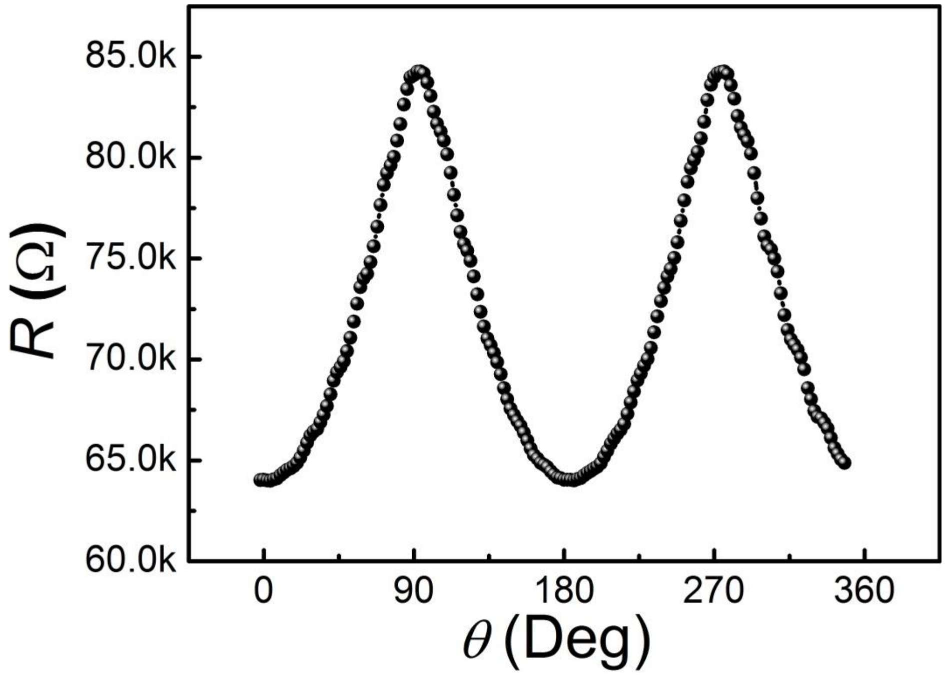

Supplementary Figure 2 | AMR of graphene on SiC. Angular-dependent resistance of the device measured at 10 K under a magnetic field of 14 T rotated from an out-of-plane (θ=0°, 180°) to an in-plane configuration (θ=90°, 270°) along the direction of the current flow.

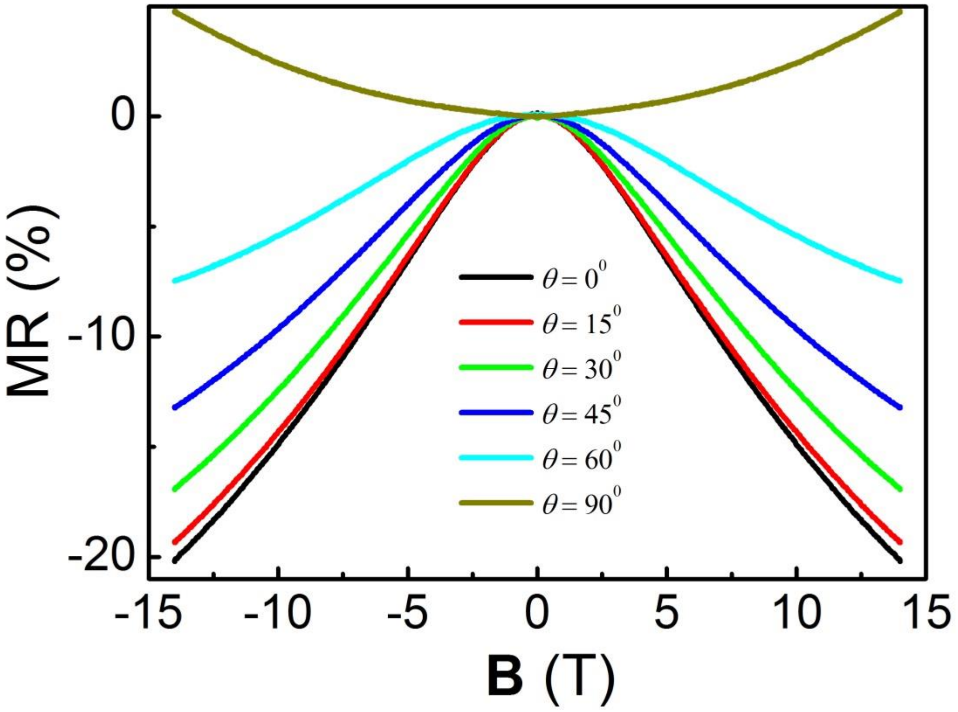

Supplementary Figure 3 | Angular dependent MR of graphene on SiC. MR measured at 10 K for a selection of field orientations, from the magnetic field in an out-of-plane (perpendicular to graphene plane, θ = 0°) to an in-plane (aligned with the current, θ = 90°) configuration, where θ is the angle between the direction of the magnetic field and the direction normal to the graphene plane.

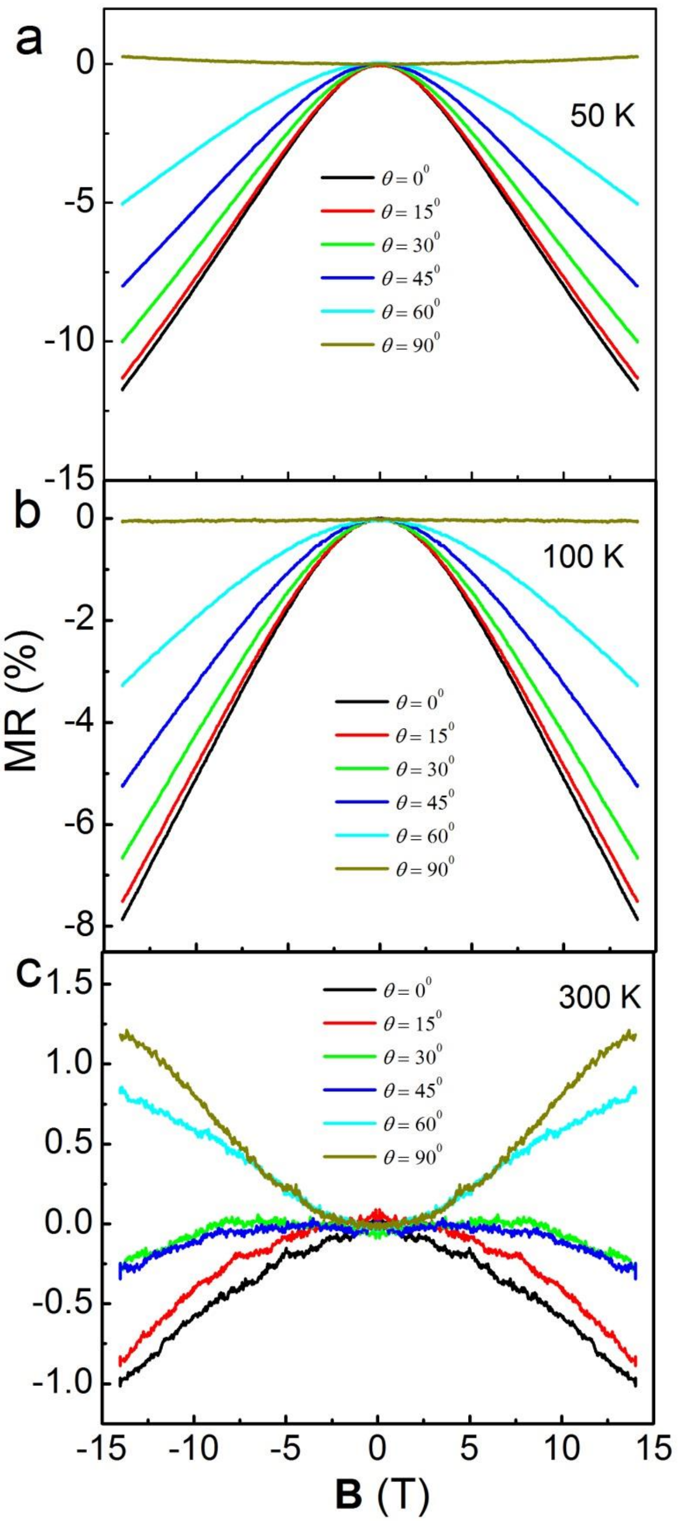

Supplementary Figure 4| Angular dependent MR at various temperatures. MR curves measured at (**a**) 50, (**b**) 100 and (**c**) 300 K with magnetic fields rotated from an out-of-plane (θ=0°) to an in-plane configuration (θ=90°) along the direction of the current.

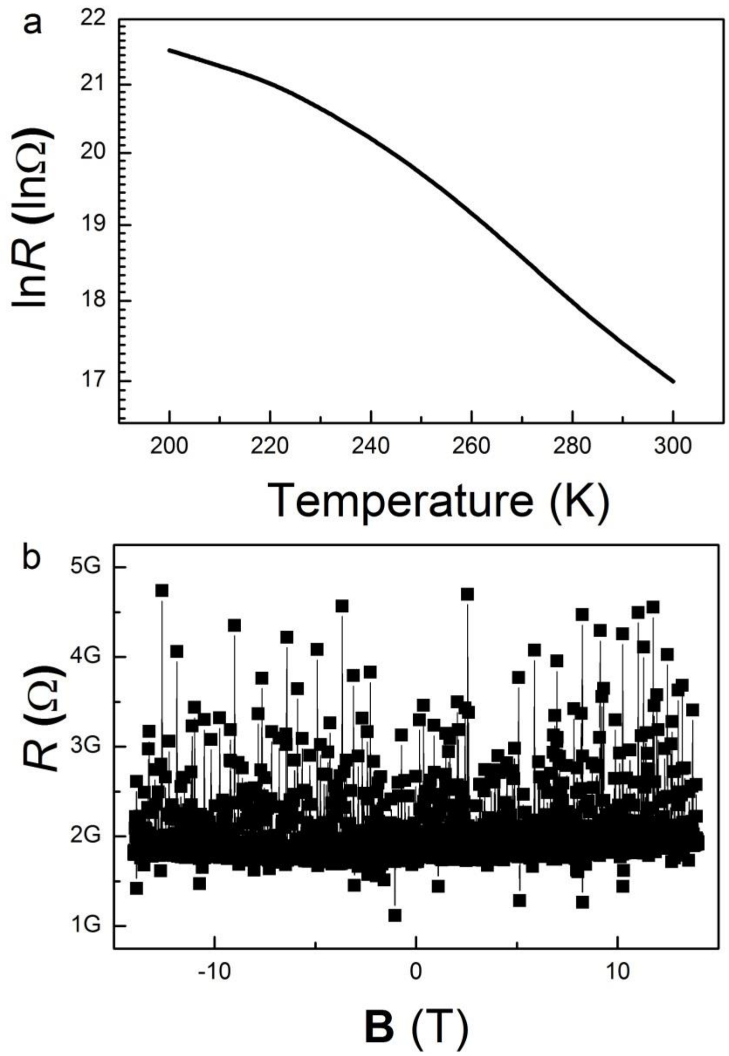

Supplementary Figure 5 | *R*-*T* and MR of bare SiC substrate. (a) *R*-*T* of the bare SiC substrate used for the growth of graphene. (b) MR of bare SiC substrate measured at 200 K.

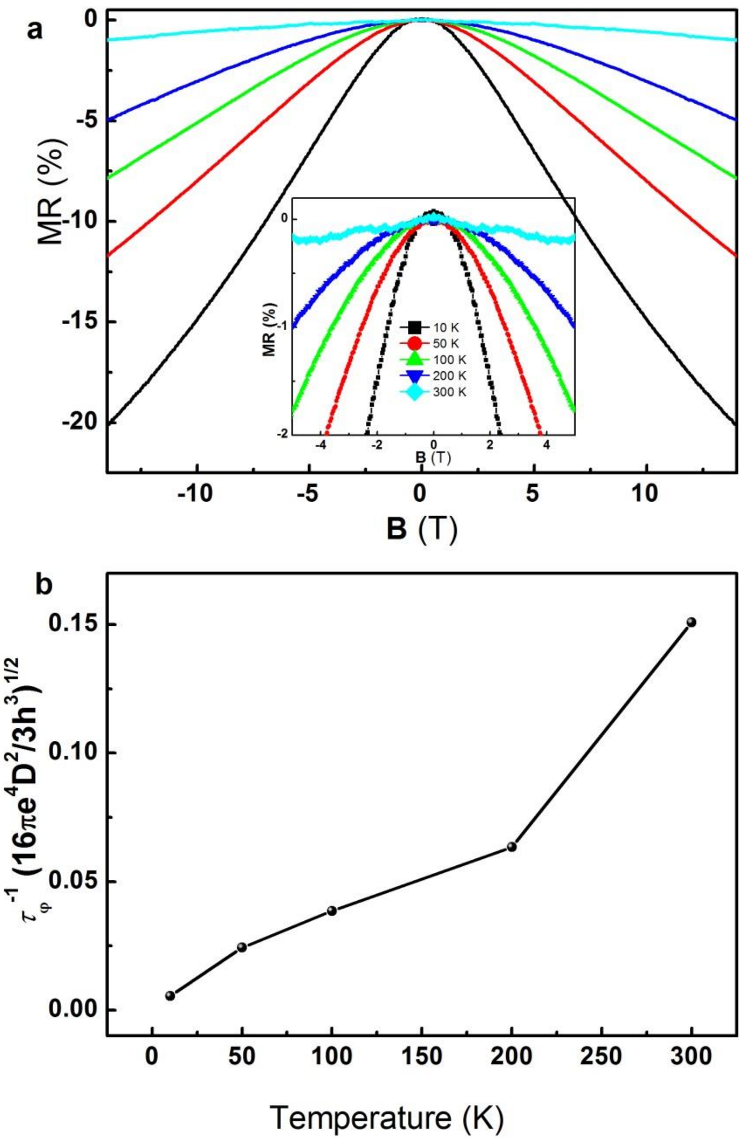

Supplementary Figure 6 | Dephasing rate of graphene at zero field. (a) MR curve of graphene measured at various temperatures with the magnetic field applied perpendicular to the graphene plane. (b) Dephasing rate as a function of temperature at zero applied magnetic field, extracted from magnetoresistance.

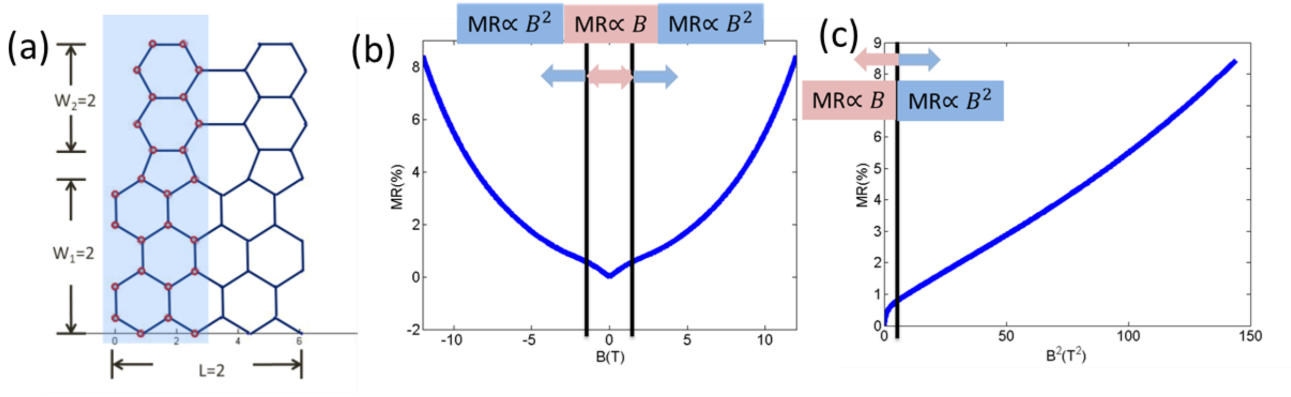

Supplementary Figure 7 | Calculated MR of graphene with one NB. (a) Schematic drawing of unit cell used, where L is the length of the NB, W_1 is the width of armchair region and W_2 is the width of zigzag region. (b) and (c) Calculated MR with a model of 3-7-7. With small parallel magnetic field (**B**) the MR is linear with respect to **B** but in most range of field strength is proportional to square of the field **B**.

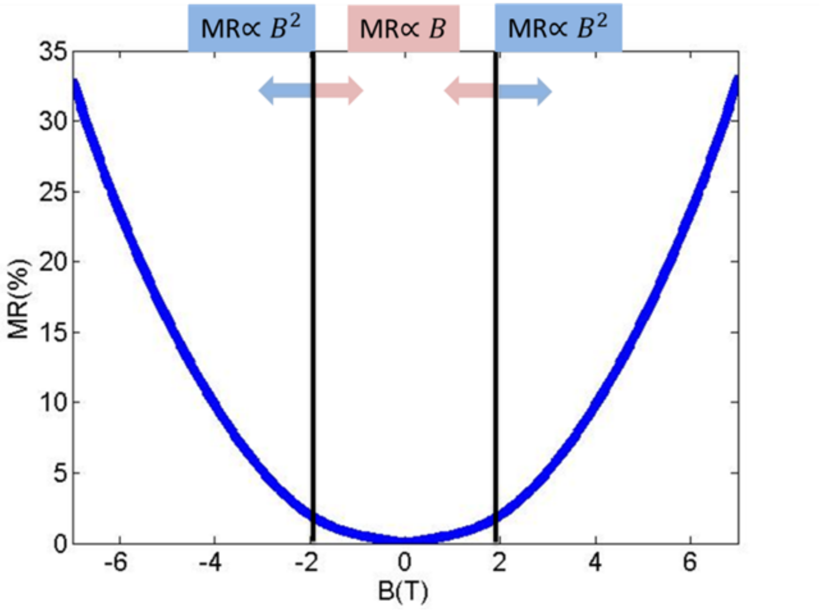

Supplementary Figure 8| Calculated MR of graphene with one NB for larger size sample.

For larger samples such as 30-7-7, the calculated MR ratio tends to match the experimental result better. The estimated effective mass is about 1.13X10^{-2} m_0 and the corresponding NB is about 10.39 nm long.

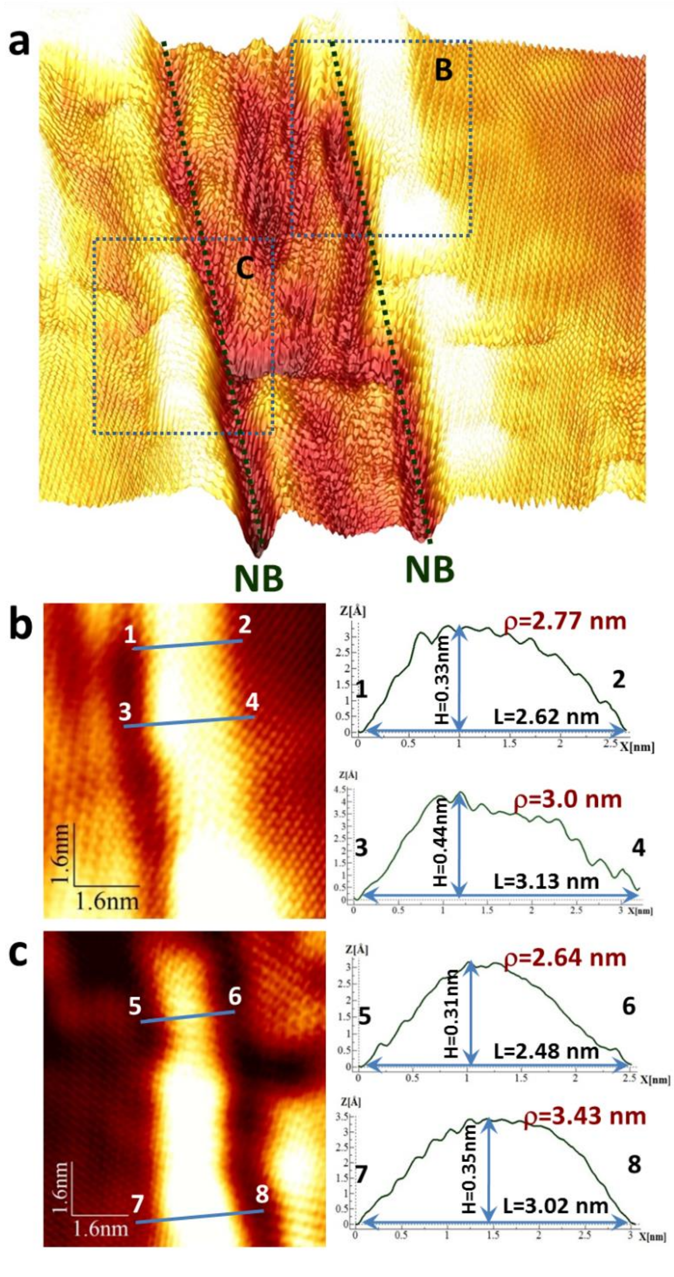

Supplementary Figure 9 | Radii of curvature at the ripples. (a) A quasi-3D 20×22 nm^2 atomically resolved STM image of graphene/SiC(001) containing three nanodomains and two boundaries (NB). Dashed rectangles B and C indicate the surface areas shown in panels (b) and (c). (b, c) Atomically resolved STM images of two nanodomain boundaries and cross-sections 1-2, 3-4, 5-6 and 7-8 of the ripples. The radii of curvature at the ripples (ρ), estimated using the formula ρ=H/2 + L^2/(8H), are shown on each profile.

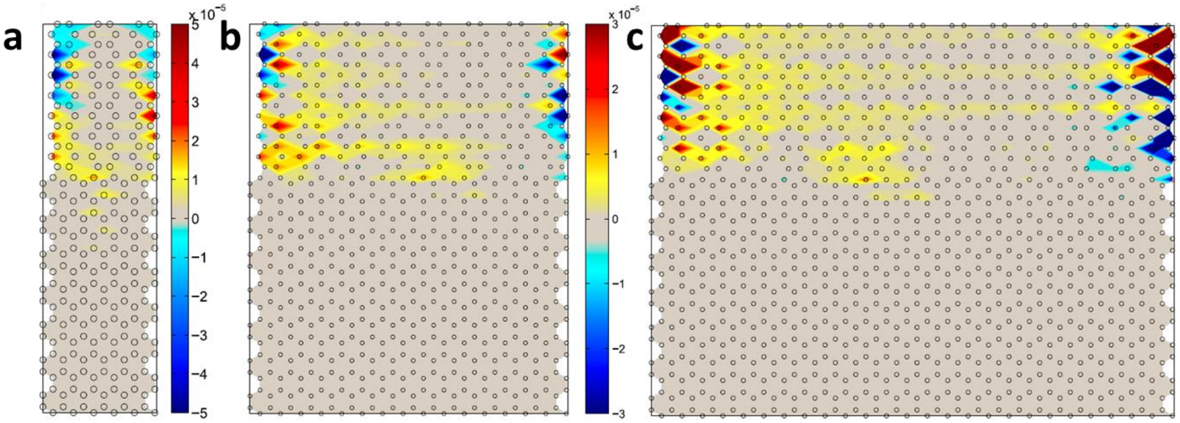

Supplementary Figure 10 | Calculated spin density distribution. Spin density distribution in z-direction demonstrates that the spin filtering effect is qualitatively independent of NB length, shown for SOC at the NB of 0.1 meV for three different lengths (a) 3 unit cells (b) 8 unit cells (c) 13 unit cells.

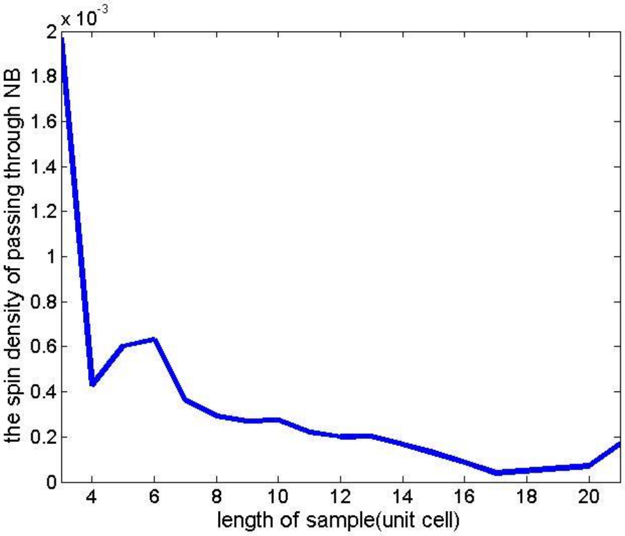

Supplementary Figure 11 | Spin density as a function of NB length. Calculated spin density as a function of length of sample.

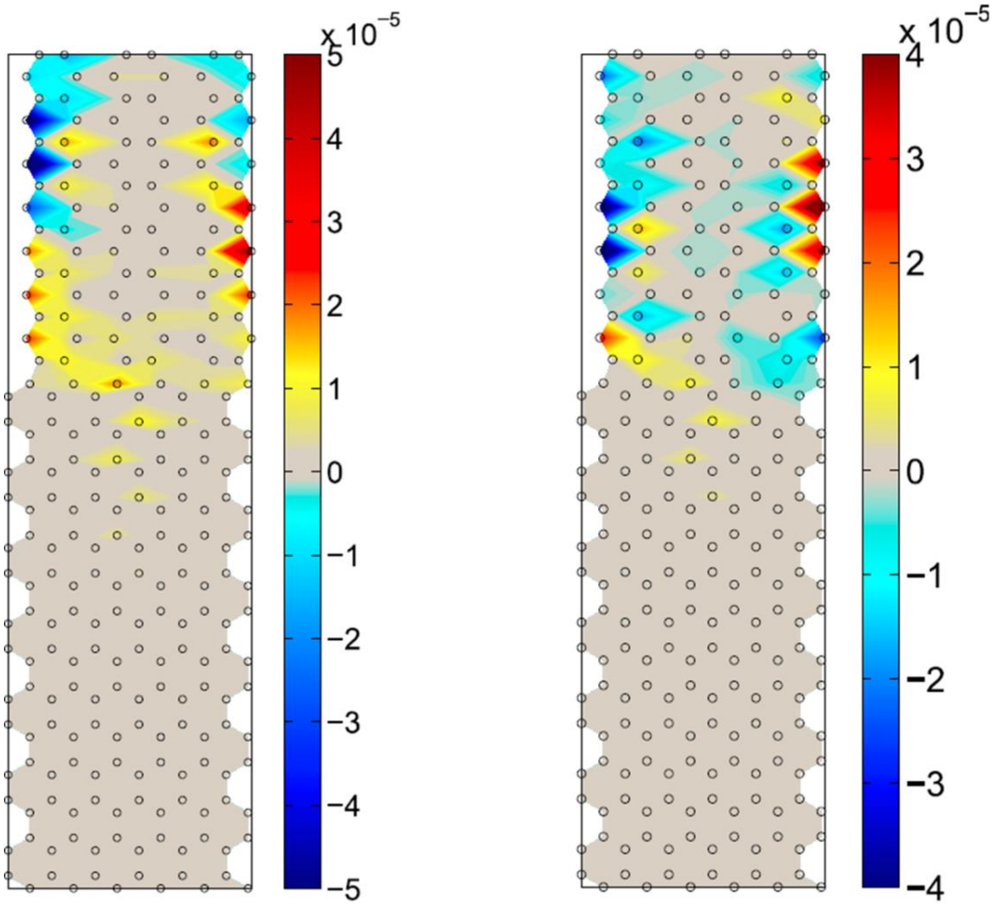

Supplementary Figure 12 | Calculated spin density for two types onsite energy. Disordered NB, for SOC of 0.1 meV. The figure on the left assumes the onsite energy is 0. While on the right there is a random on-site energy between -0.5 *t* to 0.5 *t*, where *t* is the hopping energy.

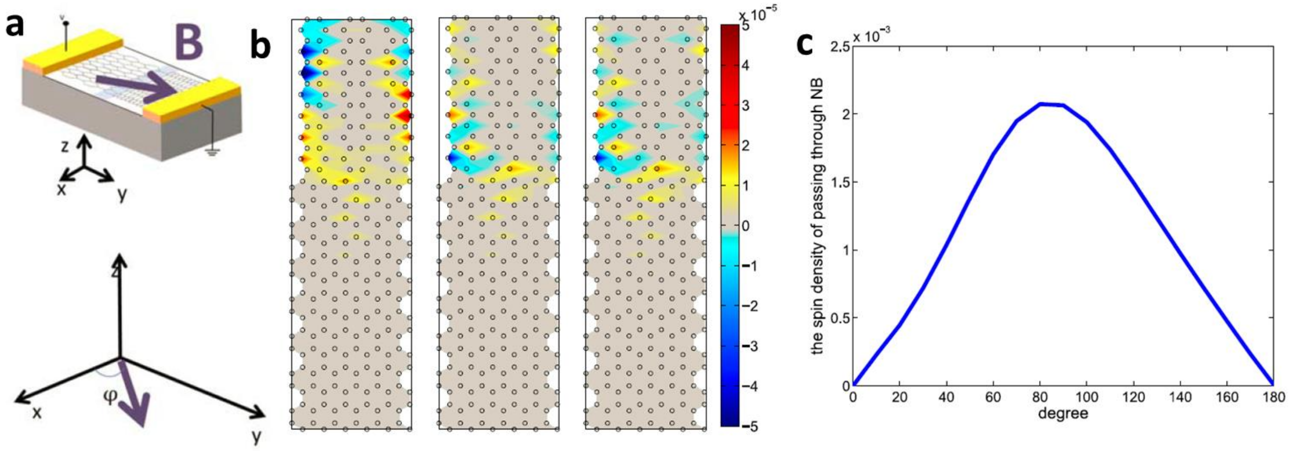

Supplementary Figure 13 | Spin density distribution with field angles. (a) Shows the relative orientation of the field with respect to the NB. (b) Shows the spin density distribution in z-direction, which demonstrates that the spin filtering is qualitatively unaffected by the orientation of the field, shown for SOC at the NB of 0.1 meV for a variety of angles. (c) Shows how the spin density passing through the NB changes with field angle.

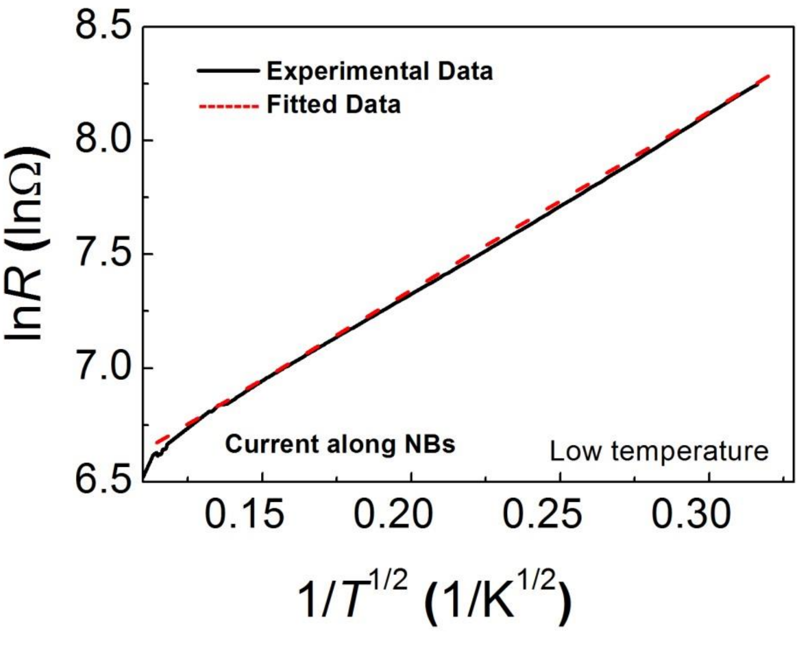

Supplementary Figure 14| *R-T* for voltage applied along NBs. The log of resistance ln(R) fitted as a straight line with respect to $1/T^{1/2}$ at low temperature, showing a better fit when the voltage is applied along the NBs suggesting better 1D transport.

Supplementary Notes

Supplementary Note 1: Characterization

Supplementary Fig. 1a shows the electron reflectivity curves measured during LEEM experiments of five different surface regions separated by micrometer-scale distances. Three distinct minima were observed in all the reflectivity curves proving the uniformity of the trilayer graphene coverage on the studied SiC(001) sample. A typical Raman spectrum of the graphene on a SiC substrate taken with an excitation photon energy of 2.33 eV is shown in Supplementary Fig. 1b. Three peaks were observed at 1376.2, 1528.7 and 1611 cm^{-1}, which correspond to the D, G and D′ bands of graphene, respectively. The D band is due to the double resonant Raman scattering induced by defects. An important feature is the 2D or G′ peak at 2740 cm^{-1} which originates from a second order process, involving two in-plane transverse optical modes near the K point. In the case of graphene, the broadening of the 2D band can originate from the band splitting of graphene (*i.e.* the presence of multiple layers), strain, or a variation in carrier doping. In our experiment, the 2D band can be fitted with 6 Lorentzians with FWHMs ranging from 21 up to 24.5 cm^{-1}, further suggesting that the grown graphene is trilayer in nature. At the same time, the blue-shift in the position of the 2D band can be related to the presence of defects and mechanical strain within the trilayer, which were clearly observed during STM characterization.

Supplementary Note 2: Contribution of SiC substrate

Supplementary Fig. 5 shows the *R*-*T* and MR of a SiC substrate used for the growth of graphene. *R*-*T* characterization indicates that the bare substrate has a resistance of 10 MΩ at room temperature and 10 GΩ at 200 K. For trilayer graphene on SiC, the resistance is around 3K Ω at room temperature and 80 KΩ at 10 K. The resistance of the bare substrate is at least 3 orders of magnitude higher than that of graphene. Thus, the contribution of the SiC substrate can be ignored.

Moreover, no MR effect was observed for bare SiC at 200 K. Thus, it can be ruled out that the observed MR effect is due to the SiC substrate.

Supplementary Note 3: Formation of alloys on SiC substrate

Our systematic studies of the graphene/SiC(001) systems by various surface science techniques (scanning tunneling microscopy, low energy electron microscopy, photoelectron spectroscopy) verified that the formed structures were free of contaminants, and consisted of quasi-freestanding few-layer graphene overlayers weakly interacting with the substrate. Graphene on SiC(001) possesses all the properties of quasi-freestanding graphene.[1,2] The graphene was prepared in an ultra-high vacuum environment, ensuring a negligible exposure to external contaminants. Alloy phases could not be detected either with electron spectroscopy techniques, averaging over millimeter-scale areas, or local probe methods providing information about the surface structure on atomic scale. Even single-atomic impurities were rarely observed on the surface during our atomic resolution STM studies, proving the uniformity and quality of the graphene nanodomain structure throughout the millimeter-sized graphene/SiC/Si(001) samples.

Supplementary Note 4: Spin scattering at magnetic/nonmagnetic impurities

Supplementary Fig. 6a shows the MR curve of graphene measured at various temperatures with a magnetic field applied perpendicular to the graphene plane. A negative MR is observed, indicating a possible weak localization effect. We extracted the dephasing rates at various temperatures using equation (2) in Ref. 3 and summarized them in Supplementary Fig. 6b. It shows a non-linear response with temperature. Thus, spin scattering at magnetic impurities can be ruled out. We would like also to stress that it is known that weak localization usually disappears at 100K. In our case, the negative MR is seen to persist even at room temperature. Moreover, since the spin dephasing time due to impurities is in the order of 10 ps,[3] and if we consider a moderate diffusion

constant of D ~ 100 cm^2s^{-1},[4] we can estimate that the mean free path is in the range of about a few tens of nm to hundreds of nm (consistent with those estimated in Supplementary Fig. 6b), which is larger than the average distance between NBs in our sample (~ 30 nm). Therefore, even if magnetic impurities existed and if they were to contribute to the resistance change, we expect the NBs still have much larger influence. Besides this, according to our NEGF calculations which consider the NB arrangement, in-plane and out-of-plane fields produce positive and negative MRs respectively, and this consistent with the experimental data.

We would like also to stress that our atomic resolution STM studies show that the graphene nanodomain structure is uniform throughout millimeter-sized graphene/SiC/Si(001) samples. Almost no single-atomic impurities were observed on the surface. The observed negative MR is mainly due to the diffuse scattering at the NBs. The domain size of our trilayer graphene is from 5 nm up to 30 nm, as shown by STM characterization. Thus, scattering at the NBs will be a dominant factor that restricts the carrier mean free path. Under the influence of a strong normal magnetic field, the trajectories of charge carriers in the graphene plane will be curved from straight lines into arcs with a radius of the magnetic length $r = \sqrt{\frac{\hbar c}{e\mathbf{B}}} = 26\ \mathrm{nm}/\sqrt{\mathbf{B}\ [\mathrm{T}]}$. As the strength of the magnetic field (**B**) increases, the radius shrinks with the inverse of the square root of the **B** field. The radius is less than 26 nm for magnetic fields stronger than 1 T, which is approximately of the same order as the domain size. As the scattering probability is proportional to the ratio between the cross section encountered by the magnetic length and the nanodomain area, a larger magnetic field produces a smaller cross section, which is less likely to be scattered by the NBs and hence a smaller resistance is observed. The resistance change is proportional to the cross section and thus a **B**-dependent linear increase in negative MR is observed, as in Supplementary Fig. 6a. Thus, it is reasonable to suggest the explanation for the positive in-plane MR comes from the intrinsic properties of the NBs themselves.

Supplementary Note 5: MR according to Kubo-Greenwood formula

In general, according to the Kubo-Greenwood formula, the conductivity of our graphene under an in-plane field can be described as:

$$\sigma = -\int dE\sigma(E)\frac{\partial f}{\partial E}, \quad (1)$$

where $\sigma(E)$ is the conductivity as a function of energy, $f(E) = \frac{1}{\exp(\frac{E-\mu}{K_B T})+1}$ is the Fermi function, and μ is the chemical potential. Due to the Zeeman effect, the latter is replaced by $\mu \rightarrow \mu \pm \mu_B \mathbf{B}$, depending on the spin projection. Expanding Supplementary Equation (1) in terms of magnetic field, we get:

$$\Delta\sigma(B) = -\frac{(\mu_B \mathbf{B})^2}{2}\int dE\sigma(E)\frac{\partial^3 f}{\partial E^3} \approx \frac{(\mu_B \mathbf{B})^2}{2}\frac{\partial^2\sigma(E=\mu)}{\partial\mu^2}, \quad (2)$$

where the last equality assumes a degenerate electron gas ($k_B T << \mu = E_F$). According to the Drude formula, $\sigma(\mu) = \frac{e^2 v_F^2}{2}N(\mu)\tau(\mu)$ where $N(\mu)$ is the density of states, v_F is the Fermi velocity and τ is the mean-free path.[4] For massless Dirac fermions in pristine graphene, $v_F = const$, $N(\mu) \propto \mu$ and, for most typical long-range scattering mechanisms, such as Coulomb impurities, frozen ripples, and resonant impurities, $\tau(\mu)$ is more or less proportional to μ, which can give a positive $\Delta\sigma(\mathbf{B})$.[4] The positive MR under an in-plane magnetic field here implies a negative $\Delta\sigma(\mathbf{B})$ that requires a sublinear behavior $\sigma(\mu) \propto \mu^\alpha$ with $0 < \alpha < 1$.

Supplementary Note 6: Sizes and radii of curvature of the ripples

Considering the ripples at NBs as the top parts of cylinder arcs, we estimate the radius of curvature from the height (H) and lateral size of the ripples (L) (determined from the cross-sections of atomically resolved STM images) using the geometrical formula for a circular segment, $\rho = H/2 +$

$L^2/(8H)$. According to the STM data (*e.g.*, Supplementary Fig. 9), the radii of curvature of the ripples in our graphene were in the range of 2.5 - 4.5 nm which is reasonably close to the values (1-3 nm) explaining spin-orbit coupling in carbon nanotubes.

Supplementary Note 7: Effect of disordering

We investigated the following through modeling: variation of length; disorder within a single NB; and the orientation of the magnetic field. From the attached figures we can conclude the relative strengths of the spin-filter and confinement effects are only marginally influenced by these factors and also that the fundamental phenomenon are still observed.

Considering first the change in NB length, we modeled lengths from 3 unit cells to 21 unit cells in the *x*-direction. The length of one unit cell is about 0.52 nm, so we are considering sample sizes between 1.56 nm to 10.72 nm. As seen from Supplementary Fig. 10, qualitatively the behavior remains the same, regardless of width. The full range is plotted in Supplementary Fig. 11, and while the spin density passing through the NB may change, it still only easy for the spin up electrons to pass through the NB. Thus the spin filtering behavior is preserved.

Secondly we consider disordering within a NB. For the disordering case we changed the on-site energy at a NB. The on-site energies at the NB were randomly chosen, in a range between $-0.5t$ to $0.5t$, where t is the hopping energy. However as can be seen from Supplementary Fig. 12, only spin up electrons are seen to readily pass through the NB, and again the spin filtering effect is preserved.

Thirdly we investigated the effect of relative magnetic field orientation. We show in Supplementary Fig. 13, that while there is a change in the density of the charges passing through the NB as the angle is varied from 0 – 180 degrees, the filtering behavior is again preserved.

Thus, due to the consistent filtering behavior of the NBs regardless of variation of length; disorder within NB; and the orientation of the magnetic field, and due to the large scale uniformity of the

graphene, it would appear that adding up disorder should not pose a significant risk of diminishing the performance at the length scales we probe.

Supplementary references

1. Chaika, A. N. *et al.* Continuous wafer-scale graphene on cubic-SiC(001). *Nano Res.* **6** 562-570 (2013)

2. Chaika, A. N. *et al.* Rotated domain network in graphene on cubic-SiC(001). *Nanotechnology* **25** 135605 (2014)

3. Lara-Avila, S. *et al.* Influence of Impurity Spin Dynamics on Quantum Transport in Epitaxial Graphene. *Phys. Rev. Lett.* **115**, 106602 (2015)

4. Katsnelson, M. I. Graphene: Carbon in Two Dimensions, Cambridge: Cambridge Univ. Press, 2012.
