# Supplementary material for: Large positive in-plane magnetoresistance induced by localized states at nanodomain boundaries in graphene
Source: Nat Commun. 2017 Feb 15;8:14453. doi: 10.1038/ncomms14453 (PMC5316875; doi:10.1038/ncomms14453)
Supplement: Peer Review File [file ncomms14453-s2.pdf]

Reviewers' Comments:

Reviewer #1 (Remarks to the Author)

Report on -
Han-Chun Wu et al., All Carbon Spin Filter Induced by Localized States at Nanodomain Boundaries in Graphene

Authors investigate the in-plane magnetoresistance of graphene grown on cubic -SiC (001). These samples consists of nanodomain boundaries sandwich zig-zag structures between adjacent ripples of large curvature. The magnetoresistance measurements showed large positive in-plane positive magnetoresistance from 5 - 35 %, which is attributed to localized states at the nanodomain boundaries. Authors claim it to be a spin filtering effect.

However, I find such claims to be premature at this stage and not well supported from the experimental data and information provided in the manuscript. Therefore, I recommend major revision of the manuscript addressing the following issues before any decision can be made.

1. The electrical conductivity of the cubic SiC substrate. Authors have not presented any data for the cubic SiC substrate used for growth of graphene, which can be conducting and can contribute to the transport measurements presented here. I doubt the strange temperature dependence and different exponents of B dependence in MR signal is coming from the temperature dependence and MR of cubic SiC background conduction. Authors should provide control experiments to rule out these effects by performing transport measurements only on SiC substrate used here.
2. Positive MR can come also from alloys, which should be discussed and ruled out.
3. From the Hall bar measurements, carrier mobility, concentration should be provided and discussed.
4. From the magnetoresistance measurements, weak localization/anti-localization effects should be presented in details. The results should be discussed in terms of diffusion length, different scattering mechanisms in their graphene in terms of magnetic and non-magnetic impurity scattering, inter and intra valley scattering. For example see: PRL 115, 106602 (2015).
5. What is the stacking and order of the tri-layer graphene (ABA...) in this case and their influence in MR measurements?
6. Is the STM measurements are made with constant current? How to rule out any artifacts in the measured data showing ripple sizes ?
7. Fig. 4c. How to distinguish this from Efros-Shklovskii gap, the gap in the density of state by interaction of localized electrons.
8. In the introduction, authors should cite papers on spin transport in CVD graphene (doi:10.1038/ncomms7766) and proximity induced magnetism in graphene (Phys. Rev. Lett. 114, 016603, 2015).

Reviewer #2 (Remarks to the Author)

The authors investigate the in-plane magnetoresistance behavior of tri-layer graphene samples grown on cubic SiC reporting a change in the magnetoresistance characteristics as a function of temperature. They attribute this effect to the 1D confinement vs. the 2D delocalization (by temperature induced hopping through nanodomain boundaries) of the charge carriers, combined with a spin splitting due to the spin orbit coupling induced by the ripples present at the domain wall edges.

Although the strong dependence of the magnetoresistance on the temperature could be an interesting observation, the explanation provided by the authors is rather speculative, as:

1. The authors don't provide enough information about the structure of the domain boundaries (e.g. the role of the grain boundary located within the domain wall). 1D localized sates and

magnetic moments can be explained by the presence of a grain boundary alone (see e.g. Sci. Rep. 5, 11744). Therefore, it is not possible to discuss the role of nanodomain boundaries, without considering the role of the grain boundaries within.

2. The interpretation of the authors is based on a substantial spin-orbit coupling in graphene, which they attribute to the curvature ("ripples") at the domain wall edges. The validity of this assumption is still an open question. However, the main problem with it is that the curvature based on the provided STM data (Fig 1d) seems much lower to me than estimated by the authors. Since in the z direction the height is about 0.3 nm, the local radius of curvature realistically should be about 10 nm, which is not consistent with the large SOC value in small radius (1-3 nm) carbon nanotubes.

The limited understanding of the observed effect is also reflected by the lack of a consistent interpretation for the individual (a couple of) NBs. It is also not entirely clear how the disorder of individual GB could average out, as claimed by the authors, to display a more perfect 1D channel like behavior. In transport measurements, I would rather expect that such disorder will add up resulting in less and less ideal behavior as more and more NB are probed. In some cases it cannot be excluded that some disorder effects could cancel out, but the authors should provide a valid argument why it is the case in their experiment..

In conclusion, while the reported magnetoresistance effect could be of interest, the present interpretation is not well enough supported by the experimental data provided in the manuscript. Such high profile claims as "a spin filter effect is achieved without external fields and persists at room temperature", and "we show that graphene with NBs has localized states and large spin-orbital interaction at the ripples" are not justified.

Reviewer #3 (Remarks to the Author)

In this manuscript, Wu and co-authors present an experimental and theoretical study of grapheme spin filter induced by localized states nanodomain boundaries. For the system graphene grown on cubic SiC(001) wafers, the authors show that localized states on nanodomain boundaries result in a positive in-plane magnetoresistance and a spin filter effect at room temperature.

In my opinion, the work is comprehensive and presents interesting potential application of graphene material. It is an excellent paper to publish on Scientific Report or Physical Review B. If the following points could be clarified, it could be reconsidered for publication on this journal:

1. According to the paper, the nanodomain structure in graphene is the dominant reason for the magnetoresistance and spin filter effect. Nanodomain could be viewed as a kind of line-defect in graphene. The problem is whether such nanodomain could be controlled in material growth or it just occurred incidentally.

2. What is the direction of in-plane magnetic field, parallel or perpendicular to transport direction? I suggest it is better to give a schematic view in the figure.

3. It is better to show the unit of magnetic field in Figs 2, 3, 4.

4. There are some typos in the manuscript, such as "spin life times" in the first sentence of Abstract.

Reviewer #1 (Remarks to the Author):

Report on -

Han-Chun Wu et al., All Carbon Spin Filter Induced by Localized States at Nanodomain Boundaries in Graphene

Comments:

Authors investigate the in-plane magnetoresistance of graphene grown on cubic -SiC (001). These samples consists of nanodomain boundaries sandwich zig-zag structures between adjacent ripples of large curvature. The magnetoresistance measurements showed large positive in-plane positive magnetoresistance from 5 - 35 %, which is attributed to localized states at the nanodomain boundaries. Authors claim it to be a spin filtering effect.

However, I find such claims to be premature at this stage and not well supported from the experimental data and information provided in the manuscript. Therefore, I recommend major revision of the manuscript addressing the following issues before any decision can be made.

1. The electrical conductivity of the cubic SiC substrate. Authors have not presented any data for the cubic SiC substrate used for growth of graphene, which can be conducting and can contribute to the transport measurements presented here. I doubt the strange temperature dependence and different exponents of B dependence in MR signal is coming from the

temperature dependence and MR of cubic SiC background conduction. Authors should provide control experiments to rule out these effects by performing transport measurements only on SiC substrate used here.

Response:

Thank you very much for your good suggestion. We have conducted transport measurements you suggested. **Figure R1** shows the R-T and MR of a SiC substrate used for growth of graphene. R-T characterization indicates that bare substrate has a resistance of 10M Ω at room temperature and 10 G Ω at 200 K. For trilayer graphene on SiC, the resistance is around 3K Ω at room temperature and 80K Ω at 10 K. The resistance of the bare substrate is at least 3 orders of magnitude higher than that of graphene. Thus, the contribution of the SiC substrate can be ignored. Moreover, no MR effect was observed for bare SiC at 200 K. Thus, it can be ruled out that the observed MR effect is due to the SiC substrate. We have clarified this point in the revision.

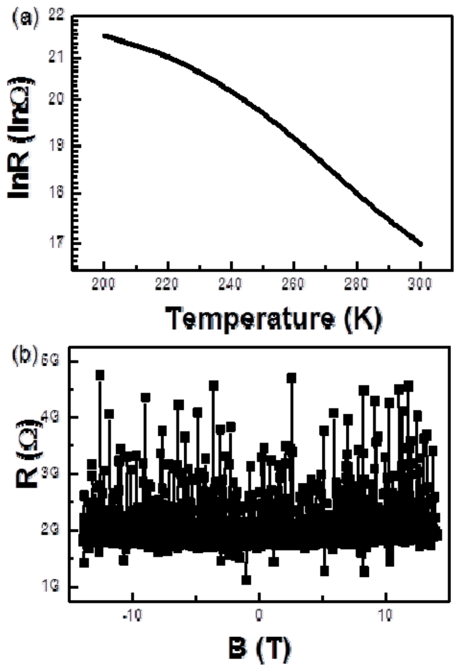

Figure R1. (a) R-T of the bare SiC substrate used for the growth of graphene. (b) MR of bare SiC substrate measured at 200 K.

The resistance of the SiC is now discussed in the main text and the supporting matierals. See from page 7 of the supporting material and lines 9 to 12 page six of the main text.

"**It can also be ruled out that any of these features arise due to electrical contributions by the silicon carbide substrate or the formation of alloys on the silicon carbide substrate. As the resistance of the bare SiC is at least three orders of magnitude greater than the graphene, the influence of the substrate can be ignored**."

Comments:

2. Positive MR can come also from alloys, which should be discussed and ruled out.

Response:

Thank you very much for your good suggestion. We agree with you that alloys can result in a positive MR. However; the formation of alloys on our silicon carbide samples can be ruled out. Our systematic studies of the graphene/SiC(001) systems by various surface science techniques (scanning tunneling microscopy, low energy electron microscopy, photoelectron spectroscopy) verified that the formed structures were free of contaminants, and consisted of quasi-freestanding few-layer graphene overlayers weakly interacting with the substrate. As demonstrated in our previous works (Nano Res. 6 (2013) 562; Nanotechnology 25 (2014) 135605), graphene on SiC(001) possesses all the properties of quasi-freestanding graphene. The graphene was prepared in an ultra-high vacuum environment, ensuring a negligible exposure to external contaminants. Alloy phases could not be detected either with electron spectroscopy techniques, averaging over millimeter-scale areas, or local probe methods providing information about the surface structure on atomic scale. Even single-atomic impurities were

rarely observed on the surface during our atomic resolution STM studies, proving the uniformity and quality of the graphene nanodomain structure throughout the millimeter-sized graphene/SiC/Si(001) samples.

This issue is now discussed in the main text and the supporting matierals. See from page 7 of the supporting material and lines 12 to 16 on page six of the main text.

“**Moreover, our systematic studies of the graphene/SiC(001) systems by various surface science techniques have verified that no alloys were formed on the SiC substrate and the formed structures consisting of quasi-freestanding few-layer graphene overlayers weakly interacting with the substrate, were free of contaminants.**”

Comments:

3. From the Hall bar measurements, carrier mobility, concentration should be provided and discussed.

Response:

Thank you for pointing out this issue. The electron mobility in our sample is about 250 cm^2/Vs at 10 K and 60 cm^2/Vs at 300 K, and the corresponding current densities are 2 x 10^{12} cm^{-2} at 10 K and 2 x 10^{13} cm^{-2} at room temperature. For trilayer graphene prepared by mechanical exfoliation, electron mobility is around 1000 cm^2/Vs. Thus, the presence of NBs decreases the electron mobility of graphene prepared on SiC and significant numbers of electrons are confined to NBs at low temperature. We have discussed this issue in the revision. See lines 7 to 14 page 5 of the main text.

“From Hall measurements it was determined that the electron mobility in our samples is about 250 cm^2/Vs at 10 K and 60 cm^2/Vs at 300 K, and the corresponding current densities are 2 x 10^{12} cm^{-2} at 10 K and 2 x 10^{13} cm^{-2} at room temperature. This value for electron mobility is notably less than the approximately 1000 cm^2/Vs observed for trilayer graphene prepared by

mechanical exfoliation.[41] It can be concluded that the presence of NBs decreases the electron mobility of graphene prepared on SiC and suggests that a significant numbers of electrons are confined to the NBs at low temperature."

Comments:

4. From the magnetoresistance measurements, weak localization/anti-localization effects should be presented in details. The results should be discussed in terms of diffusion length, different scattering mechanisms in their graphene in terms of magnetic and non-magnetic impurity scattering, inter and intra valley scattering. For example see: PRL 115, 106602 (2015).

Response:

Thank you very much for suggesting this discussion and we agree with you that magnetic/nonmagentic impurities could cause spin (flip or non-flip) scattering such that the magnetoresistance would change. **Figure R2a** shows the MR curve of graphene measured at various temperatures with the magnetic field applied normal to graphene plane. A negative MR is observed, indicating a possible weak localization effect. According with your suggestions, we have extracted the dephasing rates at various temperatures using equation 2 from PRL 115, 106602 (2015) and summarized them in **Figure R2b**. It shows a non-linear response with temperature. Thus, spin scattering at magnetic impurities can be ruled out. We would like also to stress that it is known that weak localization usually disappears at 100K. In our case, the negative MR is seen to persist even at room temperature. Moreover, since the spin dephasing time due to impurities is in the order of 10 ps,[46] and if we consider a moderate diffusion constant D ~ 100 cm^2/s,[47] we can estimate that the mean free path is in the range of about a few tens of nm to hundreds of nm (consistent with those estimated in **Figure R2b**), which is larger than the average distance between NBs in our sample (~ 30 nm). Therefore, even if magnetic impurities (if they exist) contribute to the resistance change, we expect the NBs still have much larger influence. Besides this, according to our NEGF calculations which consider the

NB arrangement, in-plane and out-of-plane fields produce positive and negative MRs respectively, and this consistent with the experimental data.

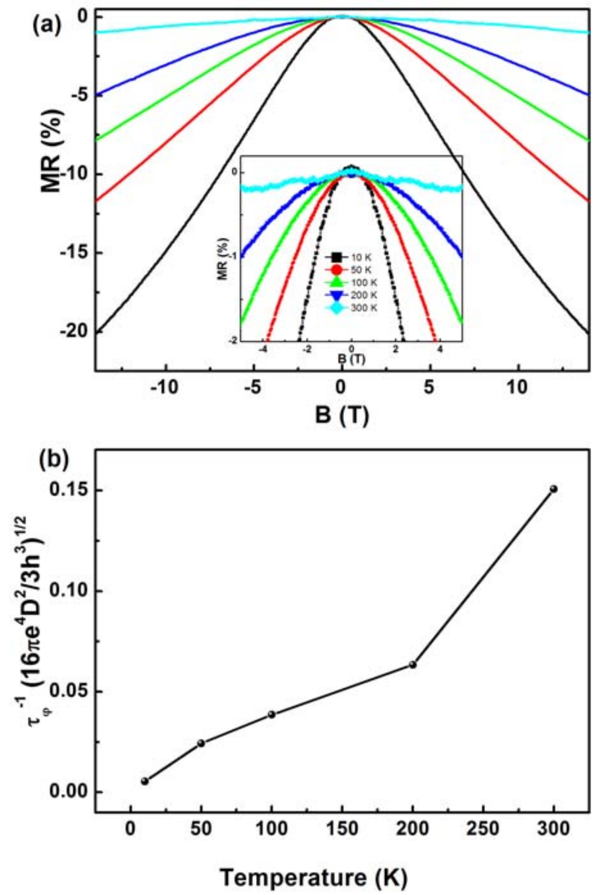

Figure R2: (a) MR curve of graphene measured at various temperatures with the magnetic field applied perpendicular to graphene plane. (b) Dephasing rate as a function of temperature at zero applied magnetic field, extracted from the magnetoresistance.

We would like also to stress that our atomic resolution STM studies show that the graphene nanodomain structure is uniform throughout millimeter-sized graphene/SiC/Si(001) samples. Almost no single-atomic impurities were observed on the surface. Moreover, as demonstrated in our previous works (Nano Res. 6 (2013) 562; Nanotechnology 25 (2014) 135605), graphene on SiC(001) possesses all the properties of quasi-freestanding graphene. The observed negative

MR is mainly due to the diffuse scattering at the NBs. The domain size of our trilayer graphene is from 5 nm up to 30 nm, as shown by STM characterization. Thus, scattering at the NBs will be a dominant factor that restricts the carrier mean free path. Under the influence of a strong normal magnetic field, the trajectories of charge carriers in the graphene plane will be curved from straight lines into arcs with a radius of the magnetic length $\mathrm{r} = \sqrt{\frac{\hbar c}{eB}} = 26\ \mathrm{nm}/\sqrt{B\ [\mathrm{T}]}$. As the strength of the magnetic field (*B*) increases, the radius shrinks with the inverse of the square root of the B field. The radius is less than 26 nm for magnetic fields stronger than 1 T, which is approximately of the same order as the domain size. As the scattering probability is proportional to the ratio between the cross section encountered by the magnetic length and the nanodomain area, a larger magnetic field produces a smaller cross section, which is less likely to be scattered by the NBs and hence a smaller resistance is observed. The resistance change is proportional to the cross section and thus a B-dependent linear increase in negative MR is observed, as in **Figure R2(a)**.

This matter is now discussed in the main text and in the supporting material and the appropriate references have been cited. See from page 8 in the supporting material and from line 16 on page 6 to line 3 on page 7 in the main text

"**Another possibility is that spin scattering at magnetic/nonmagnetic impurities could results in a positive MR. Fig. S6a shows the MR curve of graphene measured at various temperatures with the magnetic field applied normal to the graphene plane. A negative MR is observed, indicating a possible weak localization effect. We have extracted the dephasing rates at various temperatures and summarized them in Fig. S6b.[46] It shows a non-linear response with temperature. Thus, spin scattering at magnetic impurities can be ruled out.[46] Moreover, since the spin dephasing time due to impurities is on the order of 10 ps and if we consider a moderate diffusion constant D ~ 100 cm^2/s,[47] the mean free path is in the range of about a few tens of nm to hundreds of nm (consistent with those estimated in**

Figure S6b), which is larger than the average distance between NBs in our sample (~ 30 nm). Therefore, even if magnetic impurities were to contribute to the resistance change (if they exist at all), we expect the NBs will still have a much larger influence. We would like also to stress that our atomic resolution STM studies show that the graphene nanodomain structure is uniform throughout millimeter-sized graphene/SiC/Si(001) samples. Almost no single-atomic impurities were observed on the surface. In addition to this, using our non-equilibrium Green's function (NEGF) calculations which consider the NB arrangement, we will show that in-plane and out-of-plane fields produce positive MR and negative MRs respectively, and this consistent with the experimental data."

Comments:

5. What is the stacking and order of the tri-layer graphene (ABA...) in this case and their influence in MR measurements?

Response:

Thank you very much for this interesting question. Calculations of the electronic spectra of trilayer graphene with different stacking orders and a comparison with experimental angle resolved photoemission data were conducted in our previous studies (ACS Nano 9 (2015) 8967). According to the studies, the stacking order of the graphene overlayer exhibits an ABA-type structure (Bernal stacking). Since angle resolved photoelectron spectroscopy provides us information about the electronic structure on the millimeter-scale, we believe that sharp dispersions typical of ABA-stacked trilayer graphene (ACS Nano 9 (2015) 8967) prove there are negligible contributions from other stacking order variants, which thus cannot have a substantial influence on the MR properties of our graphene. Moreover, our NEGF calculations show that even for monolayer graphene, a similar spin filter effect can be observed.

Comments:

6. Is the STM measurements are made with constant current? How to rule out any artifacts in the measured data showing ripple sizes?

Response:

All the STM measurements were carried out in the constant current mode because of the nanometer-scale roughness of the graphene overlayer which is typical of freestanding graphene (Nature Materials 6 (2007) 858). The ability to resolve the atomic structure of the nanodomain boundaries and the honeycomb structure inside the nanodomains proves the extreme sharpness of the tips used and rules out possible artifacts related to the tip structure and a finite sized tip apex. The atomic resolution images of the graphene overlayer allow us to measure precisely the lateral dimensions of the individual ripples (comparing them with the well-known lattice parameter of graphene). Large area STM images of graphene/SiC(001) frequently revealed single atomic steps on the SiC substrate below the graphene trilayer. As demonstrated in our previous works, the heights of the steps were generally very close to the well-known lattice parameter of cubic-SiC (e.g., see Fig. 1 in Nano Res. 6 (2013) 562 and Fig. 2 in Nanotechnology 25 (2014) 135605). Therefore, we can exclude substantial mistakes in determining the lateral and vertical sizes of the ripples in our high resolution STM experiments.

To further illustrate the possible influence of tip size, in **Figure R3** we compare three STM images created using the tip-surface dilation function in WSxM software to the original experimental STM image of the nanostructured graphene on SiC(001). This Figure shows that details at the atomic scale are lost when increasing the radius of curvature of the tip apex from 0 to 1.0 nm, however, the ripples' sizes remain almost unchanged. Therefore, we believe that such artifacts can be excluded considering the high resolution STM data shown on Figures 1 and **Figure R3(a)**.

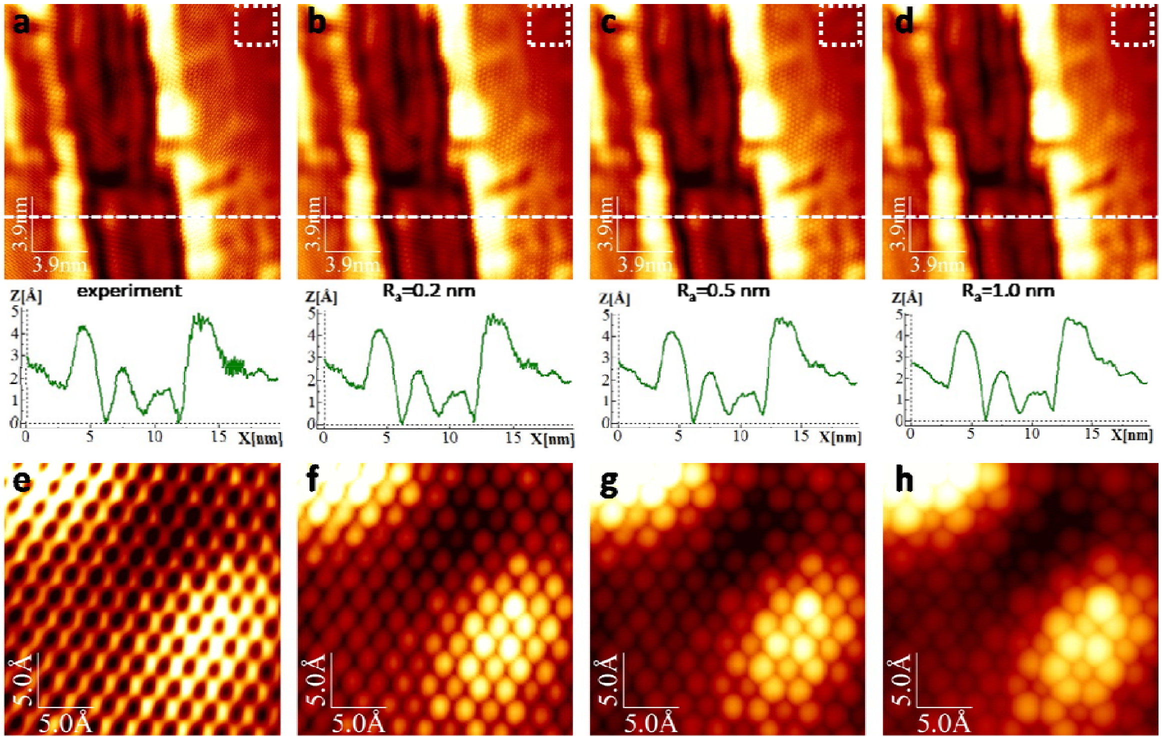

Figure R3: Original experimental STM image (a) of the nanostructured graphene on SiC(001) compared to images created using the tip-surface dilation function in WSxM software for varying simulated tip radii (b-d). Corresponding magnified images of the area within white 25Å × 25Å boxes are presented in e-h, showing clearly that the atomic hexagonal lattice cannot be resolved with a non-perfect tip. In addition, cross-sections along the dotted lines show that definition in the z-direction is lost with larger tip sizes, however the overall scale of the ripples remains constant.

Further information on the characterization of sizes and the radii of the ripples is now provided on page 13 of the supporting material.

Comments:

7. Fig. 4c. How to distinguish this from Efros-Shklovskii gap, the gap in the density of state by interaction of localized electrons.

Response:

Efros-Shklovskii variable-range hopping (ES-VRH) has the form R~ exp[(T_0/T)]^(1/2) which is the same as that for Mott VRH at one-dimension (1D). Therefore, we cannot differentiate between the two types of VRH directly only from numerical fitting. However, typically the ES gap comes from the interaction between localized electrons within the system and thus the experimental result is independent of dimensions.

In comparison, from our tight-binding simulation, we find that a single NB by itself can indeed produce an insulating gap, similar to an ES gap, without considering the interaction between two NBs. The NB will localize the electrons within itself, forming 1D transport confinement. Therefore both an ES gap and 1D confinement from a NB can describe the low temperature behavior because both can have insulating gaps. However, the localization from the NB will confine electrons within the NB with respect to the whole graphene system but electrons can still be transported freely along the NBs. We actually have observed the 1D transport behavior both from our STM experimental data and also our transport analysis of graphene with NBs. Therefore, we conclude that the Mott VRH at 1D best describes the transport behavior of Graphene with NBs at low temperature.

Moreover, the insulating gap will start to diminish as the temperature increases, as shown in an I-V curve of our previous work (Figure 6, ACS nano 9, 8967 (2015)). Therefore, at high enough temperatures, there is no 1D confinement and the transport will develop to become 2D transport.

Comments:

8. In the introduction, authors should cite papers on spin transport in CVD graphene (doi:10.1038/ncomms7766) and proximity induced magnetism in graphene (Phys. Rev. Lett. 114, 016603, 2015).

Response:

We have added the relevant references to the revised manuscript, and they are now included as references 10 and 22.

Reviewer #2 (Remarks to the Author):

Comments:

The authors investigate the in-plane magnetoresistance behavior of tri-layer graphene samples grown on cubic SiC reporting a change in the magnetoresistance characteristics as a function of temperature. They attribute this effect to the 1D confinement vs. the 2D delocalization (by temperature induced hopping through nanodomain boundaries) of the charge carriers, combined with a spin splitting due to the spin orbit coupling induced by the ripples present at the domain wall edges.

Although the strong dependence of the magnetoresistance on the temperature could be an interesting observation, the explanation provided by the authors is rather speculative, as:

1. The authors don't provide enough information about the structure of the domain boundaries (e.g. the role of the grain boundary located within the domain wall). 1D localized sates and magnetic moments can be explained by the presence of a grain boundary alone (see e.g. Sci. Rep. 5, 11744). Therefore, it is not possible to discuss the role of nanodomain boundaries, without considering the role of the grain boundaries within.

Response:

We thank the reviewer for the raising this question. Actually, the term "nanodomain boundary" in our manuscript has the same meaning as the term "grain boundary" used by the Reviewer. The use of the term nanodomain boundary is to highlight that this type of grain boundary defines the domain size. Moreover, recently, we investigated graphene containing various types of NBs with and without spin-orbit coupling, e.g., NBs of the form 5-7 and 5-8-5, **Figure**

R4. We found that the spin filter effect can only be observed in systems containing a zigzag edge NB and with spin-orbit coupling. This further supports our experimental observations. The details of other types of NBs will be reported in later publications.

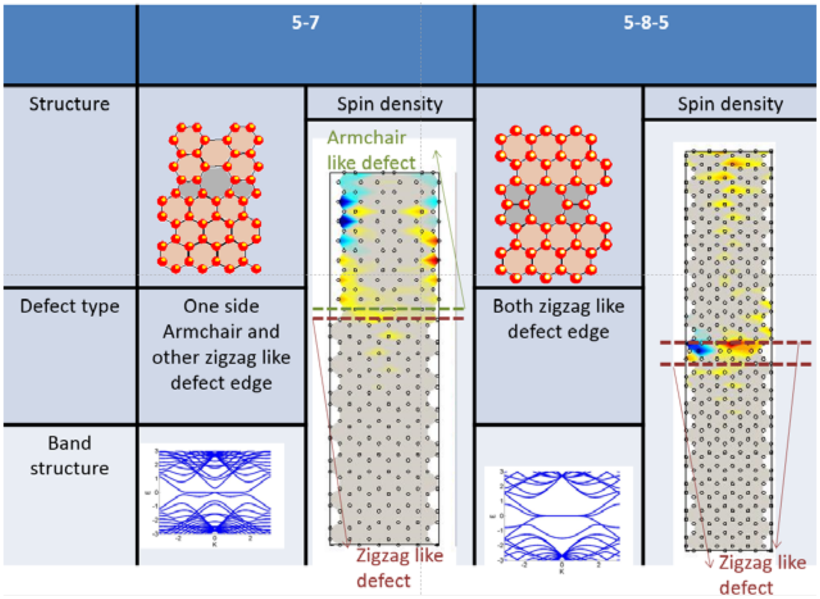

Figure R4. The effect of different NB types in graphene with SOC=0.1meV, both NB structures with zigzag edges can display spin filtering effects.

Comments:

2. The interpretation of the authors is based on a substantial spin-orbit coupling in graphene, which they attribute to the curvature ("ripples") at the domain wall edges. The validity of this assumption is still an open question. However, the main problem with it is that the curvature based on the provided STM data (Fig 1d) seems much lower to me than estimated by the authors. Since in the z direction the height is about 0.3 nm, the local radius of curvature realistically should be about 10 nm, which is not consistent with the large SOC value in small radius (1-3 nm) carbon nanotubes.

Response:

We agree with you that the SOC of CNTs and curved Graphene is still an open question, in particular the magnitude of the SOC within those systems. However, it is also well-known that curved or corrugated graphene and carbon nanotubes can strongly enhance spin-orbit coupling due to hybridization between pi and sigma bands [1,2], which has consequences like a change in the spin-relaxation time [3] and spin-splitting of edge states along the boundary [4]. Theoretical work [1,2,5] and experimental results [6, 7] all support the enhancement of SOC by curvature in graphene or CNTs.

Indeed, it is the local curvature or ripple that enhances the SOC or spin splitting, not the average curvature. The estimated SOC is about sub-meV for radii >10 nm and may be up to meV for the non-smooth local ripples, which usually have larger curvatures. We carefully analyzed the sizes and radii of curvature of the ripples using STM images providing well resolved atomic patterns both in the middle of nanodomains (far from domain boundaries) and at the boundaries. Such images can provide very precise information about the sizes of the ripples, without distortion by possible imperfections of the tip used. Considering the ripples at nanodomain boundaries as the top parts of cylinder arcs, we estimate the radius of curvature from the height (H) and lateral size of the ripples (L) (determined from the cross-sections of atomically resolved STM images) using the formula $R=H/2 + L^2/(8H)$. According to the STM data (e.g., **Figure R5**), the radii of curvature of the ripples in our graphene were in the range of 2.5--4.5 nm which is reasonably close to the values (1-3 nm) explaining spin-orbit coupling in carbon nanotubes.

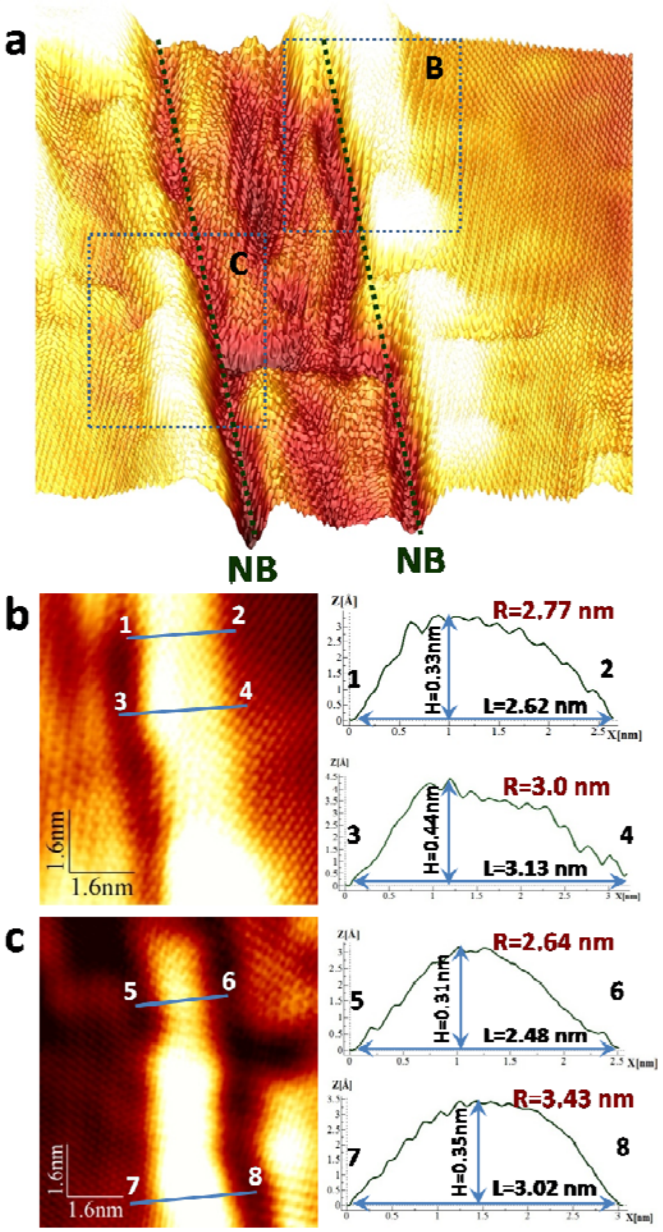

Figure R5. (a) A quasi-3D 20×22 nm^2 atomically resolved STM image of graphene/SiC(001) containing three nanodomains and two boundaries (NB). Dashed rectangles B and C indicate the surface areas shown in panels (b) and (c). (b, c) Atomically resolved STM images of two nanodomain boundaries and cross-sections 1-2, 3-4, 5-6, 7-8 of the ripples. The radii of curvature at the ripples (R) estimated using the formula $R=H/2 + L^2/(8H)$ are shown on each profile. This is now further discussed from page 13 in the supporting material.

In fact, we would like to stress that it is actually necessary for us to consider SOC or spin splitting on the NB, as it was reported that the graphene nanoribbons or graphene edges can have SOC or spin splitting up to ~7 meV [8] depending on the type of the edges. The NB considered here simultaneously displays two types of edges. There is a zigzag edge at one side of the NB while the other side is the armchair edge. Moreover, when the curvature is considered, the SOC is still stable, and may even be enhanced by the curvature. Therefore, the SOC value used in our article is not unrealistic according to previous reported articles.

Moreover, we also simulate the case for which SOC at the NB is only 0.1 meV (shown below), and found that the transport behavior actually is similar to what we reported in our article.

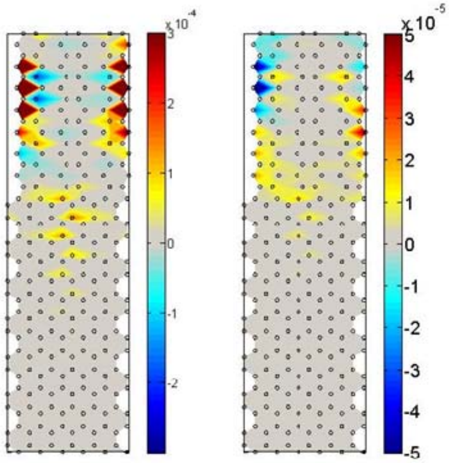

Figure R6. Left panel SOC is 8meV and right panel SOC is 0.1meV.

Therefore, the whole range of SOC strengths reported in previous publications should all show results similar to those we report here.

Comments:

The limited understanding of the observed effect is also reflected by the lack of a consistent interpretation for the individual (a couple of) NBs. It is also not entirely clear how the disorder of individual GB could average out, as claimed by the authors, to display a more perfect 1D

channel like behavior. In transport measurements, I would rather expect that such disorder will add up resulting in less and less ideal behavior as more and more NB are probed. In some cases it cannot be excluded that some disorder effects could cancel out, but the authors should provide a valid argument why it is the case in their experiment.

Response:

Thank you very much for raising this good point. We agree with you that disordering effect may exist within a regular sample, and several possible effects can be involved. As per the referee request, we investigated the following through modelling: variation of length; disorder within a single NB; and the orientation of the magnetic field. From the attached figures we can conclude the relative strengths of the spin filter and confinement effects are only marginally influenced but also that the fundamental phenomenon is still observed.

Considering first a change in NB length, we modeled lengths from 3 unit cells to 21 unit cells in the x-direction. The length of one unit cell is about 0.52 nm, so we are considering sample sizes between 1.56 nm to 10.72 nm. As seen from **Figure R7**, qualitatively the behavior remains the same, regardless of width. The full range is plotted in **Figure R8**, and while the spin density passing through the NB may change, it still only easy for the spin up electrons to pass through the NB. Thus the spin filtering behavior is preserved.

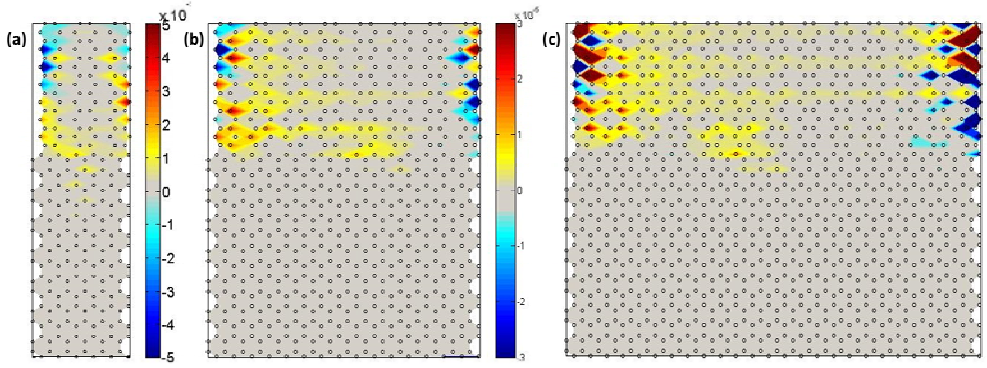

Figure R7. Spin density distribution in z-direction demonstrates that the spin filtering effect is qualitatively independent of NB length, shown for SOC at the NB of 0.1 meV for three different lengths (a) 3 unit cell (b) 8 unit cell (c) 13 unit cell.

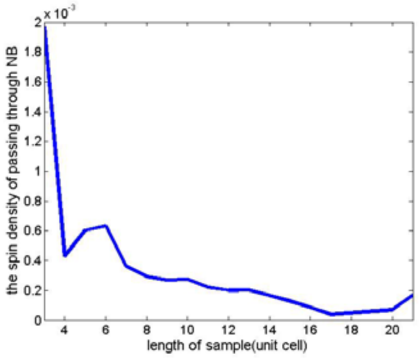

Figure R8. Spin density as a function of NB length.

Secondly we consider disordering at the NB. For the disordering case we changed the on-site energy at the boundary. The on-site energies at the NB were randomly chosen, in a range between -0.5t to 0.5t, where t is the hopping energy. However as can be seen from **Figure R9**, only spin up electrons are seen to readily pass through the NB, and again the spin filtering effect is preserved.

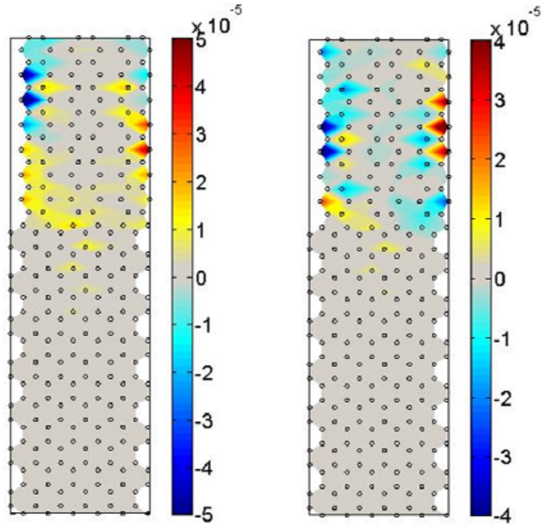

Figure R9 Disordered NB, for SOC of 0.1meV. The figure on the left assumes the onsite energy is 0. While on the right there is a random on-site energy between -0.5t to 0.5t, where t is the hopping energy

Thirdly we investigated the effect relative magnetic field orientation. We show in **Figure R10**, that while there is a change in the density of the charges passing through the NB as the angle is varied from 0 – 180 degrees, the filtering behavior is again preserved.

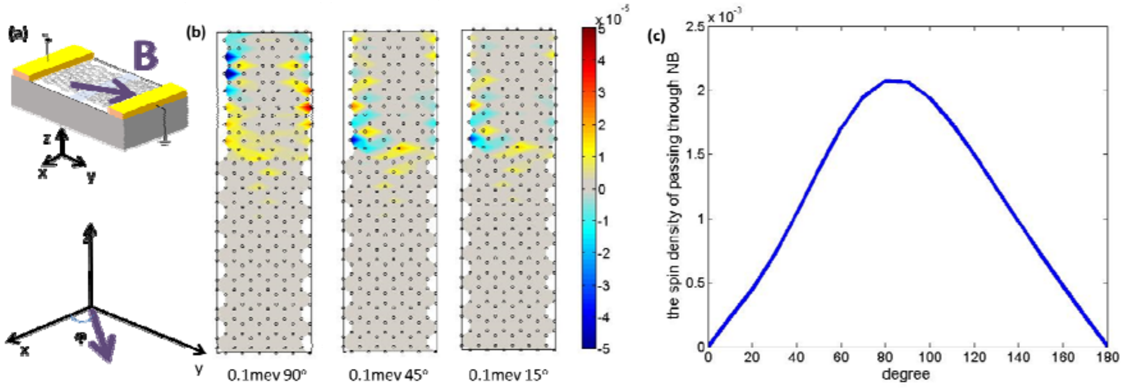

Figure R10 (a) Shows the relative orientation of the field with respect to the NB. (b) Shows the spin density distribution in z-direction, which demonstrates that the spin filtering is qualitatively unaffected by the direction orientation of the field, shown for SOC at the NB of 0.1 meV for a variety of angles. (c) Shows how the spin density passing through the NB changes with field angle.

Thus, due to the consistent filtering behavior of the NBs, regardless of changes in width, disorder within a single NB, and field orientation, and the large scale uniformity of the graphene, it would appear that adding up disorder should not pose a significant risk of diminishing the performance at the length scales we probe.

These issues are now now discussed from page 14 of the supporting material and from lines 9 to 14 on page 9 of the main text

“**It is known that disordering effects may exist within a regular sample and several possible effects can be involved. We investigated the following disordering effects: variation of length; disorder within a single NB; and the orientation of the magnetic field (Figures S10-S13). It is shown that the relative strengths of the spin-filter and confinement effects are only marginally influenced and that the fundamental phenomenon is still observed.**”

Comments:

In conclusion, while the reported magnetoresistance effect could be of interest, the present interpretation is not well enough supported by the experimental data provided in the manuscript. Such high profile claims as "a spin filter effect is achieved without external fields and persists at room temperature", and "we show that graphene with NBs has localized states and large spin-orbital interaction at the ripples" are not justified.

Response:

We believe that the changes we have made in the revision and the above response, to the concerns raised, now appropriately support our explanation that the spin transport and associated MR of Graphene with NBs are consistent with our experimental data and also with the transport theory. However we also revise our claims, adopting a more moderate descriptive way to honestly reflect our results. We have deleted the contentious claim “**Moreover, a spin filter effect is achieved without external fields and persists at room temperature.”** and have weakened the claim about **"we show that graphene with NBs has localized states and large spin-orbital interaction at the ripples".** The relevant sentence in the conclusions (line13 on page 9) now reads “*Moreover,* ***our work suggests*** *that graphene with NBs has localized states and large spin-orbit interaction at the ripples*.”

Reviewer #3 (Remarks to the Author):

Comments:

In this manuscript, Wu and co-authors present an experimental and theoretical study of grapheme spin filter induced by localized states nanodomain boundaries. For the system graphene grown on cubic SiC(001) wafers, the authors show that localized states on nanodomain boundaries result in a positive in-plane magnetoresistance and a spin filter effect at room temperature.

In my opinion, the work is comprehensive and presents interesting potential application of graphene material. It is an excellent paper to publish on Scientific Report or Physical Review B. If the following points could be clarified, it could be reconsidered for publication on this journal:

1. According to the paper, the nanodomain structure in graphene is the dominant reason for the magnetoresistance and spin filter effect. Nanodomain could be viewed as a kind of line-defect in graphene. The problem is whether such nanodomain could be controlled in material growth or it just occurred incidentally.

Response:

In our previous papers we showed that graphene grows on SiC/Si(001) substrates at high temperatures (above 1600 K) forming a nanodomain structure with ripples near the boundaries. This result was highly reproducible. The same nanodomain structure was revealed in <u>all</u> our high resolution scanning tunneling microscopy experiments conducted on a large number of samples. We should emphasize that using vicinal SiC/Si(001) substrates one can <u>controllably</u> synthesize graphene nanodomain systems with a preferential direction for nanodomain boundaries and ripples on the technologically relevant substrates suitable for electronic applications. That was demonstrated using STM for vicinal samples with a 2˚ miscut from the (001) plane (ACS Nano 9 (2015) 8967). This is also supported by our preliminary STM

data obtained on 4˚ miscut samples (unpublished) and LEEM data published recently by A. Ouerghi et al. (Appl. Phys. Lett. 101 (2012) 021603).

Comments:

2. What is the direction of in-plane magnetic field, parallel or perpendicular to transport direction? I suggest it is better to give a schematic view in the figure.

Response:

We thank the reviewer for this helpful suggestion. The in-plane magnetic field is applied parallel to the current direction. We have added a schematic to clarify the details of the experiment. See new Figure 4a.

Comments:

3. It is better to show the unit of magnetic field in Figs 2, 3, 4.

Response:

The unit of magnetic field is Tesla (T). According with the reviewer's suggestion we have shown units in figures 2, 3, and 4.

Comments:

4. There are some typos in the manuscript, such as "spin life times" in the first sentence of Abstract.

Response:

We have checked and suitably revised the language throughout the manuscript.

References:

1. D. Huertas-Hernando, F. Guinea, and A. Brataas, Spin-orbit coupling in curved graphene, fullerenes, nanotubes, and nanotube caps. *Phys. Rev. B* 74, 155426 (2006).

2. J.-S. Jeong, J. Shin, and H.-W. Lee, Curvature-induced spin-orbit coupling and spin relaxation in a chemically clean single-layer graphene. *Phys. Rev. Lett.* 84, 195457 (2011).

3. D. Huertas-Hernando, F. Guinea, and A. Brataas, Spin-Orbit-Mediated Spin Relaxation in Graphene. *Phys. Rev. Lett.* 103, 146801 (2009).

4. H. Santos, M. C. Munoz, M. P. Lopez-Sancho, and L. ChicoInterplay between symmetry and spin-orbit coupling on graphene nanoribbons. *Phys. Rev. B* 87, 235402 (2013)

5. J.-S. Jeong and H.-W. Lee, Curvature-enhanced spin-orbit coupling in a carbon nanotube. *Phys. Rev. B* 80, 075409 (2009).

6. F. Kuemmeth, S. Ilani, D. C. Ralph, and P. L. McEuen, *Nature* (London) 452, 448 (2008).

7. Steele, G. A. et al. Large spin-orbit coupling in carbon nanotubes. *Nat. Commun.* 4, 1573 (2013).

8. M. P. Lopez-Sancho and M. C. Munoz, Intrinsic spin-orbit interactions in flat and curved graphene nanoribbons. *Phys. Rev. B* 83, 075406 (2011).

Reviewers' Comments:

Reviewer #1 (Remarks to the Author)

Wu et al. have reported the observation of large positive in-plane magnetoresistance (MR) in graphene grown on SiC. The MR is interpreted to be arising from localized states at the nano domain boundaries with zig-zag structures.

I have reviewed the manuscript earlier. I am satisfied with the answers and modifications made in the manuscript. Therefore, I recommend publication of the manuscript after some minor revisions. However, another round of review is not required from my side.

I recommend to change following things.
1. Do not use "spin-filter" word in title, as there is no direct evidence from experiments. Authors can use "large positive magnetoresistance ....", for example.
2. In abstract, introduction and summary sections, I suggest to tone-down a bit, do not use "spin-filtering" and "spintronics application" word too much.

Reviewer #2 (Remarks to the Author)

Comments to the authors:

- It is still not clear to me that the "nanodomain boundary" is the same as a graphene grain boundary. I mean, ND boundaries are about 5 nm wide areas with ripples at their edges, while a graphene grain boundary is narrower and usually no ripples are seen around them. From the interpretation of the authors the ripples at the edges of such ND boundaries play an essential role in the spin filtering by conferring the spin-orbit coupling necessary in their model. However, what I meant is that previous theoretical works [e.g., the already mentioned Sci. Rep. 5, 1174] predicted that a grain boundary without curvature induced spin-orbit coupling could act as a spin filter. The authors should at least discuss this possibility for their interpretation and ideally also provide some arguments why their interpretation is more likely.

- Whether the radius of curvature of the observed ripples are enough to justify the 3.5 meV spin-orbit coupling value used by the authors for the interpretation of their measurements, I'm still not convinced. For carbon nanotubes a spin orbit coupling of 0.37 meV has been measured [Ref.6]. for a 2.5 nm radius of curvature, in good agreement with the theoretical predictions. In the present work the minimum radius of curvature claimed is about 2.6-2.7 nm, and they only occur in some restricted areas, usually the curvature radius is considerably larger. Yet, the authors take the SOC value form another paper for a 1.5 nm radius of curvature [Ref. 7] that reports an anomalously large SOC of 3.4 meV (in discrepancy with the theory). I would suggest that for a radius > 2.5nm, a more realistic value is at least one order of magnitude lower curvature induced SOC value. In their reply, the authors also claim that it is not even necessary to consider the curvature, as the graphene edges alone can give rise to large SOC. However, these calculations have been performed for graphene nanoribbons and the predictions have never been experimentally verified, in contrast to the above proposed numbers. Also the authors show in their response that even for 0.1 meV SOC the transport behavior is similar. If this is the case, then they should probably discuss the results of the main text by using a similarly small, more realistic SOC value.

- As concerning the disorder, the authors model various types of disorder and reach the conclusion that they only marginally influence the observed behavior. While this is useful to point out, it still does not answer how disorder could lead to an improvement of the 1D channel-like behavior observed when probing more boundaries.

To summarize, in my opinion the interpretation of the experimental data is still too ambiguous to recommend publication in Nature Communications.

Reviewer #3 (Remarks to the Author)

Wu and co-authors' paper "All carbon spin filter induced by localized states at nanodomain boundaries in graphene" present a interesting study on the MR of graphene on cubic-SiC(001) substrate. Combined using experimental and theoretical tools, they give a self consistent conclusion that temperature dependent positive MR was observed in graphene. It is very interesting to find positive MR in graphene, because MR effect is often negative for topological materials and honeycomb lattice is a typical platform for quantum spin Hall effect and quantum anomalous Hall effect. Therefore, I suggest the paper could be published on Nature Communication.

Response to reviewers' comments

Dear Referees:

We would like to thank you very much for your efforts and time to review our work. Your concerns and comments about the paper are much appreciated. In the revised manuscript, all changes and revisions have been highlighted by formatting the text in a Red color. For your convenience, we also include below, a point-by-point list of the corrections made in response to all your concerns.

Reviewer #1 (Remarks to the Author):

Wu et al. have reported the observation of large positive in-plane magnetoresistance (MR) in graphene grown on SiC. The MR is interpreted to be arising from localized states at the nano domain boundaries with zig-zag structures.

I have reviewed the manuscript earlier. I am satisfied with the answers and modifications made in the manuscript. Therefore, I recommend publication of the manuscript after some minor revisions. However, another round of review is not required from my side.

I recommend to change following things.

Comments:

1. Do not use "spin-filter" word in title, as there is no direct evidence from experiments. Authors can use "large positive magnetoresistance ....", for example.

Response:

Thank you very much for your good suggestion. We have implemented the suggestion you made, the title of the paper is now **Large Positive In-Plane Magnetoresistance Induced by Localized States at Nanodomain Boundaries in Graphene**.

Comments:

2. In abstract, introduction and summary sections, I suggest to tone-down a bit, do not use "spin-filtering" and "spintronics application" word too much.

Response:

Thank you very much for this good suggestion. We have removed some of the sentences referring to "spin-filtering" and "spintronics applications" in the introduction and concluding summary section, and weakened the claim in the abstract.

The abstract now reads "Our work ***may*** offer a tantalizing way to add the spin degree of freedom to graphene**.**" From the introduction we delete the claim "**for a wide range of new electronic and spintronic applications**."

In the conclusion we delete the claim "**This behavior paves a way to add a spin degree of freedom to graphene.**"

Other minor improvements have been implemented throughout the manuscript

Reviewer #2 (Remarks to the Author):

Comments:

It is still not clear to me that the "nanodomain boundary" is the same as a graphene grain boundary. I mean, ND boundaries are about 5 nm wide areas with ripples at their edges, while a graphene grain boundary is narrower and usually no ripples are seen around them. From the interpretation of the authors the ripples at the edges of such ND boundaries play an essential role in the spin filtering by conferring the spin-orbit coupling necessary in their model. However, what I meant is that previous theoretical works [e.g., the already mentioned Sci. Rep. 5, 1174] predicted that a grain boundary without curvature induced spin-orbit coupling could act as a spin filter. The authors should at least discuss this possibility for their interpretation and ideally also provide some arguments why their interpretation is more likely.

Response:

Thank you very much for your good suggestion and you are exactly correct in your description. The nanodomain boundaries in our study contain both ripples and graphene boundaries. The grain boundaries in our study consist of distorted heptagons and pentagons, and the zig-zag and armchair edges develop from them. Details can be found in our earlier report (ACS Nano, 9, 8967, (2015)). We have also clarified this issue in the revision. See from line 1 to line 4 on page 5. “**Thus, the NBs are a combination of both ripples and graphene grain boundaries. The grain boundaries in our study consist of distorted heptagons and pentagons, and zig-zag and armchair edges develop from them. Further details can be found elsewhere.”**

We agree with you that graphene boundaries with specific configurations could induce a spin-filtering effect, as reported in Sci. Rep. 5, 11744, due to the asymmetry between up and down spins. However, we would like stress that the grain boundaries in our study are quite different from those in Sci. Rep. 5, 11744. The grain boundaries in our study consist of distorted heptagons and pentagons, and the ripples are formed to relieve in-plane compressive strain. Moreover, more recent theoretical studies investigated the influence of localized magnetic moments at the edges on the transport properties and suggested that these localized magnetic moments could be screened by the polarized current under bias (J. Phys. D: Appl. Phys. 49, 015305 (2016); J. Appl. Phys. 111, 07C324 (2012)). Therefore, the spin-filtering effect in our study is expected to be mainly due to SOC as reported for other curved graphene systems. We have added this discussion in the revision and the important references have been cited.

See from line 19 to line 28 on page 9:

“Note, a recent theoretical study suggested that a graphene boundary itself with a specific configuration may induce a spin-filtering effect due to the asymmetry between up and down spins.[57] However, we would like stress that the grain boundaries in our study are quite different from those in Ref. 57. The grain boundaries in our study consist of distorted

heptagons and pentagons, and the ripples are formed to relieve in-plane compressive strain. Moreover, more recent theoretical studies investigated the influence of localized magnetic moments at the edge on the transport properties and suggested that localized magnetic moments could be screened by the polarized current under bias.[58,59] Therefore, the spin-filtering effect in our study is expected to be mainly due to SOC as reported for other curved graphene systems."

Comments:

Whether the radius of curvature of the observed ripples are enough to justify the 3.5 meV spin-orbit coupling value used by the authors for the interpretation of their measurements, I'm still not convinced. For carbon nanotubes a spin orbit coupling of 0.37 meV has been measured [Ref.6]. for a 2.5 nm radius of curvature, in good agreement with the theoretical predictions. In the present work the minimum radius of curvature claimed is about 2.6-2.7 nm, and they only occur in some restricted areas, usually the curvature radius is considerably larger. Yet, the authors take the SOC value form another paper for a 1.5 nm radius of curvature [Ref. 7] that reports an anomalously large SOC of 3.4 meV (in discrepancy with the theory). I would suggest that for a radius > 2.5nm, a more realistic value is at least one order of magnitude lower curvature induced SOC value. In their reply, the authors also claim that it is not even necessary to consider the curvature, as the graphene edges alone can give rise to large SOC. However, these calculations have been performed for graphene nanoribbons and the predictions have never been experimentally verified, in contrast to the above proposed numbers. Also the authors show in their response that even for 0.1 meV SOC the transport behavior is similar. If this is the case, then they should probably discuss the results of the main text by using a similarly small, more realistic SOC value.

Response:

Thank you very much for your good suggestion. According with your suggestion, we have now added to the discussion in the main text the results of using a smaller and more realistic SOC value of 0.1 meV. See new figure 3 and line 5 to line 10 on page 9.

"However, there are some boundary regions where the radius of curvature is greater than ~2.5nm, which would mean the SOC could be an order of magnitude weaker. To investigate the spin-dependent transport across the NBs and in particular considering the case with a weaker SOC associated with a larger radius of curvature, Fig. 3e shows the calculated spin density distribution of the device under a bias voltage of 0.4 V. The SOC is set to be 0.1 meV, to reflect the regions which have larger radii of curvature."

Comments:

As concerning the disorder, the authors model various types of disorder and reach the conclusion that they only marginally influence the observed behavior. While this is useful to point out, it still does not answer how disorder could lead to an improvement of the 1D channel-like behavior observed when probing more boundaries.

Response:

We apologies if this discussion was misleading. In the main text where 1-2 NBs are probed, the voltage is applied perpendicular to NBs. In this configuration, the electrons will tunnel through the NBs. Thus, no improvement of the 1D channel-like behavior will be observed. In contrast, if the voltage is applied along the NBs we will observed a clear 1D channel behavior. This is exactly what we observed. See Figure R1. Thus probing more boundaries provides a greater opportunity for conduction along the NB. These points are now clarified in the revision, with the addition of Figure S14 in the supporting material, see line 2 on page 11.

“The gap between the electrodes was around 28 nm, thus 1–2 NBs were measured within the gap **and the voltage was applied perpendicular to the NBs.**”

And line 20 to line 22 on page 11.

“The reason is that when only 1 or 2 NBs are measured, since the voltage is applied perpendicular to the NB, the electrons must tunnel through the NBs. If we apply voltage along NBs, a clear 1D channel behavior is observed (Fig. S14).”

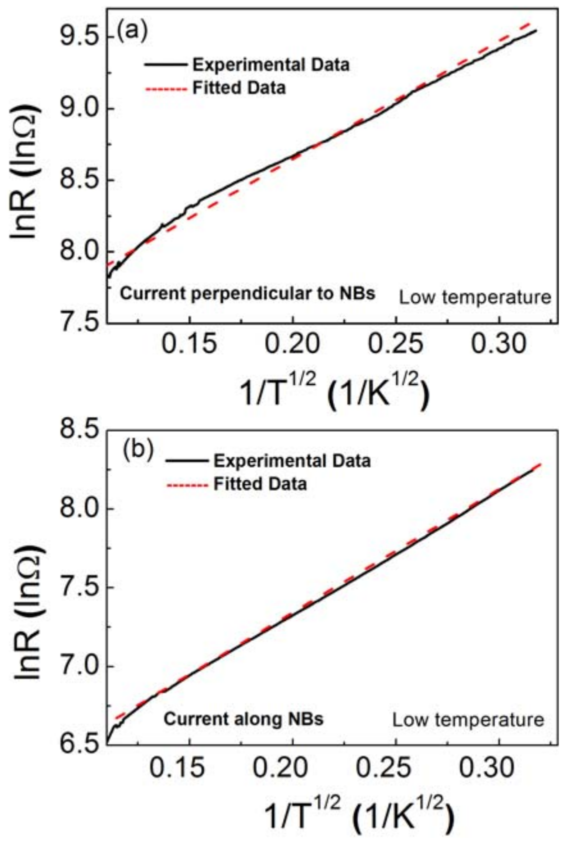

Figure R1 The log of resistance ln(R) fitted as a straight line with respect to $1/T^{1/2}$ at low temperature, showing a better fit when the voltage is applied along the NBs suggesting better 1D transport.

Comments:

To summarize, in my opinion the interpretation of the experimental data is still too ambiguous to recommend publication in Nature Communications.

Response:

We have addressed the shortcomings you highlighted and have made the changes you recommended. We hope you can now consider publication of this manuscript in Nature Communications.

Reviewer #3 (Remarks to the Author):

Comments:

Wu and co-authors' paper "All carbon spin filter induced by localized states at nanodomain boundaries in graphene" present a interesting study on the MR of graphene on cubic-SiC(001) substrate. Combined using experimental and theoretical tools, they give a self consistent conclusion that temperature dependent positive MR was observed in graphene. It is very interesting to find positive MR in graphene, because MR effect is often negative for topological materials and honeycomb lattice is a typical platform for quantum spin Hall effect and quantum anomalous Hall effect. Therefore, I suggest the paper could be published on Nature Communication.

Response:

We thank the reviewer for the helpful comments which helped improve this manuscript.

REVIEWERS' COMMENTS:

Reviewer #2 (Remarks to the Author):

The authors have addressed my concerns and suggestions adequately, and the revised manuscript comprises now a more realistic interpretation of the experimental data. I have no further major objections.

Reviewer #2 (Remarks to the Author):

Comments:

The authors have addressed my concerns and suggestions adequately, and the revised manuscript comprises now a more realistic interpretation of the experimental data. I have no further major objections.

Response:

We thank the reviewer for the helpful comments which helped improve this manuscript.
